# Photoluminescence Enhancement by Band Alignment Engineering in MoS_2_/FePS_3_ van der Waals Heterostructures

**DOI:** 10.1021/acsami.2c05464

**Published:** 2022-07-15

**Authors:** Maria Ramos, Francisco Marques-Moros, Dorye L. Esteras, Samuel Mañas-Valero, Eudomar Henríquez-Guerra, Marcos Gadea, José J. Baldoví, Josep Canet-Ferrer, Eugenio Coronado, M. Reyes Calvo

**Affiliations:** † Departamento de Física Aplicada, Universidad de Alicante, Alicante 03690, Spain; ‡ Instituto de Ciencia Molecular (ICMol), Universitat de València, Paterna 46980, Spain; § Instituto Universitario de Materiales de Alicante (IUMA), Universidad de Alicante, Alicante 03690, Spain

**Keywords:** van der Waals heterostructures, transition metal dichalcogenide monolayers, enhanced photoluminescence, band alignment engineering, optoelectronic tunability

## Abstract

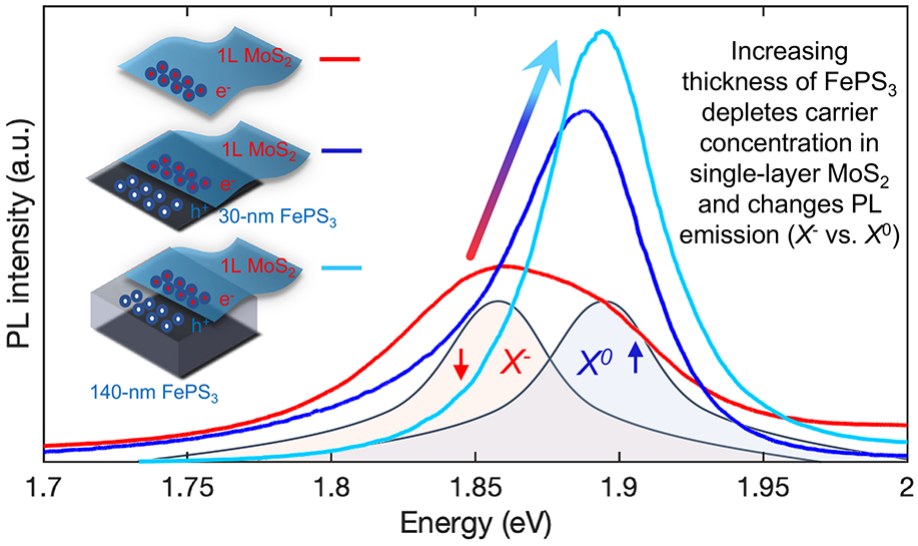

Single-layer semiconducting transition metal dichalcogenides (2H-TMDs) display robust excitonic photoluminescence emission, which can be improved by controlled changes to the environment and the chemical potential of the material. However, a drastic emission quench has been generally observed when TMDs are stacked in van der Waals heterostructures, which often favor the nonradiative recombination of photocarriers. Herein, we achieve an enhancement of the photoluminescence of single-layer MoS_2_ on top of van der Waals FePS_3_. The optimal energy band alignment of this heterostructure preserves light emission of MoS_2_ against nonradiative interlayer recombination processes and favors the charge transfer from MoS_2_, an n-type semiconductor, to FePS_3_, a p-type narrow-gap semiconductor. The strong depletion of carriers in the MoS_2_ layer is evidenced by a dramatic increase in the spectral weight of neutral excitons, which is strongly modulated by the thickness of the FePS_3_ underneath, leading to the increase of photoluminescence intensity. The present results demonstrate the potential for the rational design of van der Waals heterostructures with advanced optoelectronic properties.

## Introduction

In the past decade, two-dimensional (2D) crystals have attracted the attention of a broad community of chemists, physicists, and material scientists due to their novel mechanical, electrical, and optical properties when thinned down to just a few atomic layers.^1−7^ The direct gap and photoluminescent properties of single-layer 2H TMDs have facilitated their use as the active media of optoelectronic devices.^3,8−10^ Recently, a growing interest in a new family of 2D compounds has emerged, namely the transition metal chalcogenophosphates, with the general formula MPX_3_ (where M is a transition metal, P is phosphorus, and X is a chalcogen). MPX_3_s have been explored in terms of their antiferromagnetic phase transition,^11−19^ photo-response,^20−26^ and promising applications in spintronics.^27−32^

A fascinating perspective of the field of van der Waals materials is the endless possibilities of combining and modifying their properties by stacking different types of 2D materials in heterostructures with an atomically sharp heterointerface. When two materials with different chemical potentials are brought close, charge carriers distribute across the interface until electrostatic equilibrium is reached. This will be conditioned by the relative energy band alignment between the Fermi levels, the band onsets, and the interface quality between the two materials. The study of band alignment and charge transfer across heterostructures containing single-layer semiconducting TMDs is a powerful approach to tailor their optical and electronic properties. Hence, through the proper selection of the 2D materials, it is possible to engineer the electronic and optical properties of the materials involved.

In the case of single layers of doped semiconducting TMDs, such as MoS_2_, charge transfer has a remarkable influence on its photoluminescence emission (PL).^8−10^ Indeed, a strong enhancement of MoS_2_ PL of about 2 orders of magnitude due to charge transfer and dipolar interactions with the surroundings has been reported.^33^ However, most of the works where these observations are reported include solution-processed functionalization methods. Several works have shown how the photoluminescence yield of these 2D materials can be strongly enhanced by molecular adsorbates^34−36^ and acid treatment.^33,34^ However, in heterostructures of stacked 2D materials, charge transfer seems to be less efficient across the van der Waals barrier in terms of enhancement of the PL intensity of single-layer TMDs. While an enhancement of photoluminescence has been observed in certain heterostructures with a type I band alignment, type II band arrangements usually lead to a quench of light emission.^37−39^ Nevertheless, a fast and efficient photo-induced electron–hole dissociation into adjacent layers of a 2D heterostructure notably reduces the probabilities of exciton recombination in their constituent materials and, thus, causes a dramatic drop in the PL emission of these systems.^8−10,40−42^ Besides charge transfer, other tuning knobs for PL modulation of single-layered materials are based on strain engineering^43−46^ and the application of external back-gate electric fields.^47^

In this work, we take advantage of the strong p-type character of intrinsic FePS_3_ semiconductor and the optimal energy band alignment with n-type one-layer (1L) 2H-MoS_2_ to build vertically stacked MoS_2_/FePS_3_ heterostructures with efficient charge carrier transfer and improved light emission properties. At room temperature, the intensity of the photoluminescence of MoS_2_ increases, and the emission peak is blue shifted according to an increase of excitonic *versus* trionic recombination. Also, a remarkable increase in defect-bound exciton emission is observed at low temperatures. All these observations point to a scenario where a high proportion of the free electrons in the single-layer MoS_2_ is transferred to the FePS_3_. The efficiency of this transfer, only comparable to the adsorbates case, leads to an almost full depletion of the MoS_2_ layer, which is followed by the narrowing and raising of the PL emission. We show how these effects strongly depend on—and can be tuned by—the thickness of the FePS_3_ layer.

## Experimental Results

**Figure 1:**
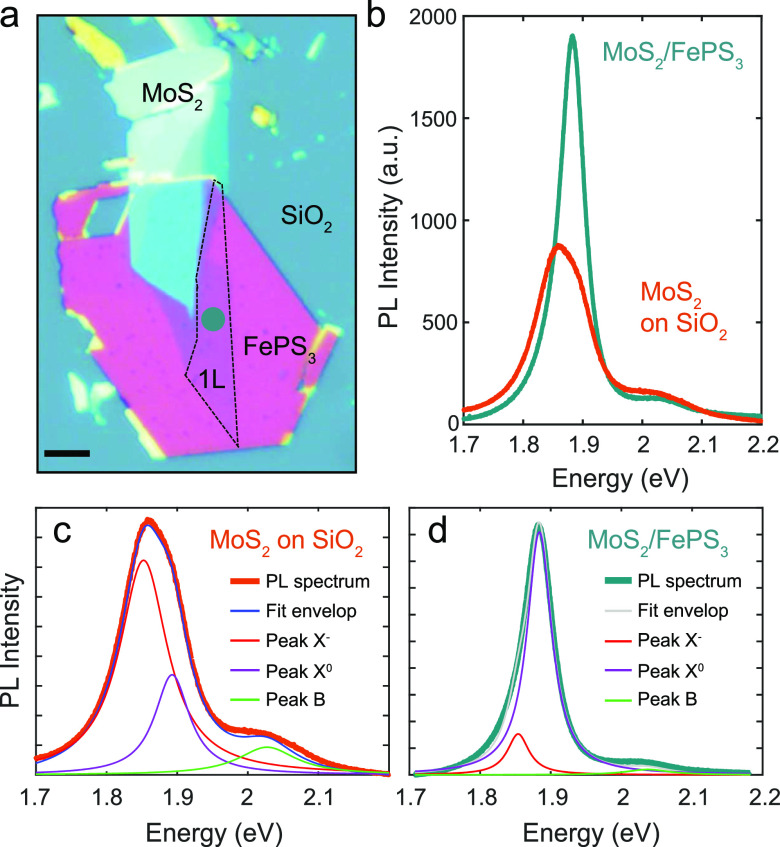
(a) Optical microscopy image of the fabricated heterostructure onto a SiO_2_/Si substrate, where the single-layer MoS_2_ (1L) is placed on top of a multilayer FePS_3_ flake. The green dot in (a) indicates the zone of the heterostructure where the spectrum shown in (b,d) was taken. The scale bar in (a) corresponds to 10 μm. (b) Photoluminescence spectra taken at the 1L-MoS_2_/FePS_3_ heterostructure (green curve), which is shown in (a), and at a control sample (orange curve), 1L-MoS_2,_ which is directly deposited on the SiO_2_/Si substrate. (c,d) Analysis of the photoluminescence spectral shapes for the as-prepared MoS_2_ monolayer and 1L MoS_2_/FePS_3_ heterostructure, respectively, assuming three peaks with Lorentzian functions: trion (*X*^–^) and neutral excitons (*X*^0^ and *B*).

Figure 1a shows one of the fabricated heterostructures consisting of a monolayer of MoS_2_ transferred onto a multilayer FePS_3_ flake (see Methods for fabrication details). The PL spectrum of the fabricated heterostructure has been measured at room temperature under a 532 nm laser excitation and compared with the PL emission of a control sample (1L MoS_2_ flake deposited directly onto the 300 nm SiO_2_/Si substrate) (Figure 1b). The two emission peaks corresponding to *A* (1.84–1.9 eV) and *B* (2.01–2.04 eV) excitons in 1L MoS_2_ are present in both PL spectra. These two emission peaks come from the recombination of electrons in the conduction band with holes in the spin–orbit split valence bands in monolayer MoS_2_.^2^ We observe that the PL spectral shape changes depending on the material where the MoS_2_ monolayer lies: the PL emission associated with exciton *A* coming from the heterostructure is brighter and narrower with an intensity about two times higher and clearly blue shifted if compared to 1L MoS_2_ directly deposited on SiO_2_. We also observe a drop in the relative spectral weight associated with exciton *B* in the heterostructure spectrum when compared to the control sample (see Supporting Information Section S3). Because this signal is considerably weaker, we focus our analysis on the evolution of the *A* exciton peak.

To unveil the origin of these PL spectral changes, we decompose the PL peak coming from exciton *A* into two subexcitonic contributions: the neutral exciton *X*^0^ (an electron and a hole bounded) and the trion or negatively charged exciton *X*^–^ (two electrons and a hole bounded).^48^ For the case of as-prepared 1L MoS_2_ (Figure 1c), the contribution of the negative trion peak (*X*^–^), located at ∼1.84 eV (red curve), prevails over the PL spectral weight of the neutral exciton (*X*^0^), located at ∼1.88 eV (purple curve). This dominant recombination mediated by trions (*X*^–^) reveals a heavily n-type doped monolayer MoS_2_, which is consistent with previous observations.^35^

In contrast to the MoS_2_ monolayer on SiO_2_, the PL emission from the heterostructure (Figure 1d) is clearly dominated by the neutral exciton peak (*X*^0^) at ∼1.88 eV, due to the presence of FePS_3_. Considering the p-type nature of FePS_3_,^20,21^ the experimental results suggest a strong charge transfer of electrons from the MoS_2_ monolayer toward the FePS_3_ flake, when these two are interfaced, and consequent depletion of the TMD layer. This experimental observation highly resembles the strong tunability and enhancement of the PL properties in monolayer TMDs *via* chemical doping.^33−36^

The equilibrium among exciton, trion, and free-electron populations in MoS_2_ can be viewed as a simple chemical reaction: *X*^0^ + *e* ↔ *X*^–^, where the rate equality of the forward and reverse reactions are described by a mass action law model.^35^

The population of the three species is then governed by a rate equation , where *N*_*X*^–^_ and *N*_*X*^0^_ are the number of trions (*X*^–^) and excitons (*X*^0^), respectively, while *K*_T_ and *n*_e_ are the rate constant for trions and the free electron density, respectively (see Supporting Information Section S4 and ref (35) for details).

The ratio between the contributions (area under the curve) of the trion (*A*_*X*^–^_) and exciton (*A*_*X*^0^_) is expected to be proportional to their respective populations in equilibrium:
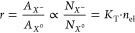
1

Similarly, the emission ratios for the heterostructure and control should then be proportional to the respective populations in the heterostructure and control samples
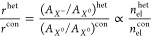
2

This provides a first estimation of the electron depletion in the 1L-MoS_2_ due to charge transfer when placed on FePS_3_. Under this assumption, the calculated relative electron concentration, (*n*_el_^con^ – *n*_el_^het^)/*n*_el_^con^, changes proportionally to (*r*^con^ – *r*^het^)/*r*^con^ for all the fabricated heterostructures and is within the range of ∼81–∼99%, reaching ∼95% for the specific heterostructure shown in Figure 1 (see Supporting Information Sections S4 and S5 for further details).

Moreover, assuming the values reported in the literature for the effective masses of electrons, excitons, and trions and the trion binding energy, as well as the radiative decay rates of trions and excitons at room temperature,^35^ we can obtain approximated values for actual electron densities in MoS_2_ in both samples (see Supporting Information Section S4 for details). Thus, the estimated electron densities of the 1L-MoS_2_ flake in the control sample and in the heterostructure are ∼4.8 × 10^13^ and ∼3.0 × 10^12^ cm^–2^, respectively. These results support our hypothesis about an efficient transfer of electrons in 1L-MoS_2_ toward FePS_3_.

While similar results have been obtained by chemical treatments or molecular physisorption on single-layer TMDs, our observation is something unique in the case of van der Waals type II heterojunctions, where typically the PL emission is strongly quenched due to spatial electron–hole separation and/or the formation of interlayer excitons.^8−10,42^

**Figure 2:**
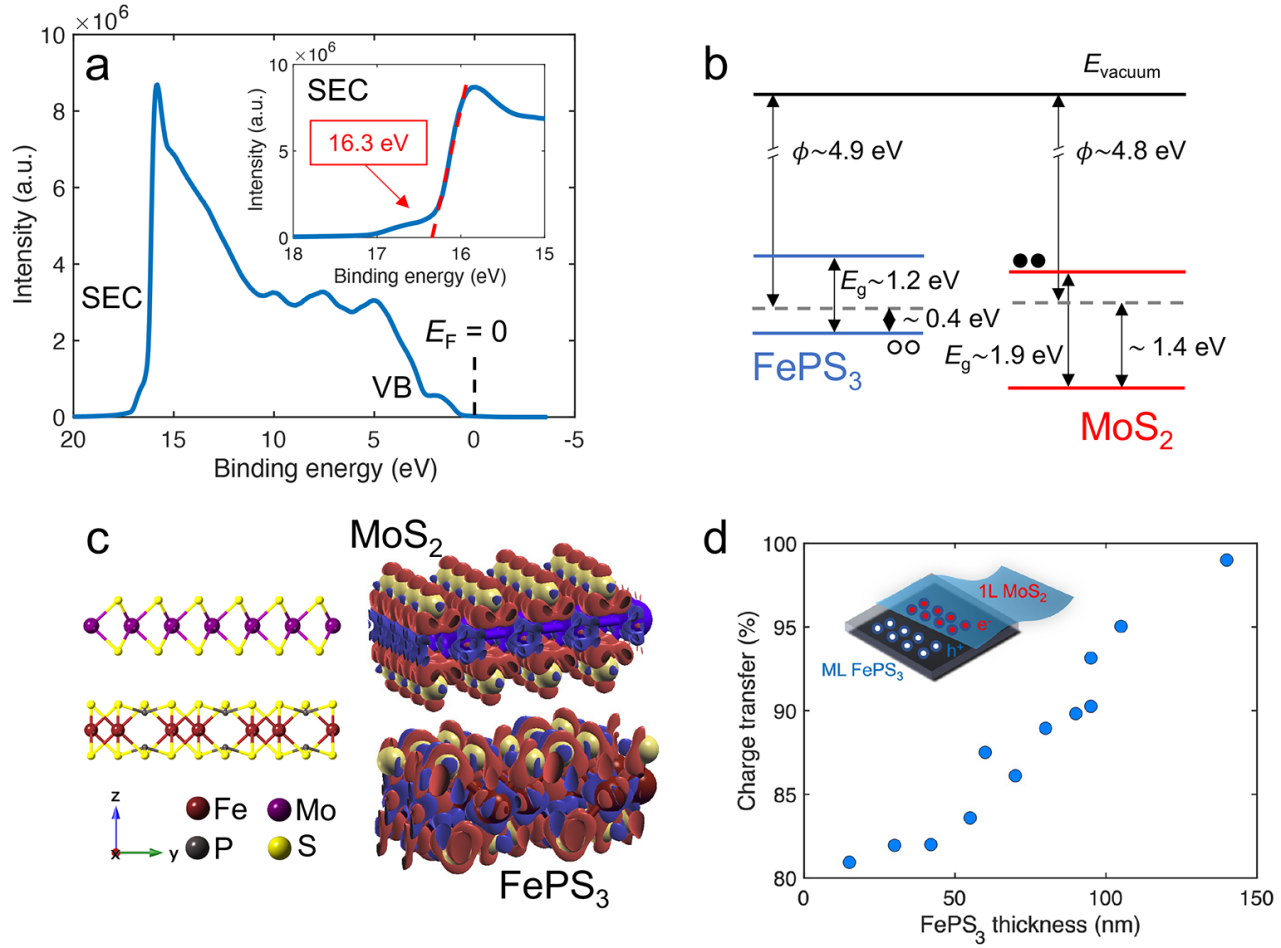
(a) UPS spectrum of bulk FePS_3_ using He I (ℏω = 21.22 eV) as a monochromatic excitation source, where emission peaks coming from valence band (VB) states and secondary electrons (SEC) can be observed. The zero binding energy indicates the Fermi level. Inset: Zoom-in of the secondary electron cut-off (SEC). (b) Experimentally estimated band diagram of the 1L MoS_2_/ML FePS_3_ junction forming a type II heterostructure. (c) Side view of the atomic MoS_2_/FePS_3_ heterointerface and its corresponding charge transfer representation using an isovalue equal to 0.05 in the XCrySDen package.^56^ The difference between the charge density and the superposition of atomic densities shows the gain (red) and depletion (blue) zones along the heterostructure, evidencing the absence of gain and depletion zones at the heterointerface. (d) Charge transfer in the heterostructure, relative to a control sample, obtained from the analysis of photoluminescence spectra as a function of the thickness of the FePS_3_ flake underneath.

To obtain further insight into the origin of this efficient charge transfer, we determine the band onset energies for FePS_3_ and 1L MoS_2_ separately. To do this, we performed ultraviolet photoelectron spectroscopy (UPS) in bulk FePS_3_. The deduction of the work function for bulk FePS_3_ is obtained from the UPS spectrum (Figure 2a) as ϕ = ℏω – SEC ≈ 4.9 eV, where ℏω is the excitation energy (He I: 21.22 eV), and SEC is the energy cut-off of the secondary electron region of the spectrum obtained from a linear fit to the data^49,50^ (see inset of Figure 2a). The work function for bulk FePS_3_ deduced in our work is slightly larger than two recently published works, reporting values of ∼4.7 and ∼4.17.^51,52^ Nevertheless, we have also obtained a similar work function value for bulk FePS_3_ through Kelvin probe force microscopy (see Supporting Information Section S7).

On the other hand, electron acceptor levels in FePS_3_ have been postulated to arise from Fe^2+^ defects.^53^ By fitting the conductivity as a function of temperature to an Arrhenius model for multilayer flakes of FePS_3_, we obtain an activation energy of ∼0.37 eV, which is in the range of the electron acceptor energies reported for bulk FePS_3_^53^ (see Supporting Information Section S8) and UPS valence band determination (see Supporting Information Section S6). Assuming this and considering that the bandgap energy of a several-layer FePS_3_ flake is ∼1.23 eV, previously deduced from photo-responsivity measurements,^21^ it is possible to draw a diagram of the energy band alignment for an FePS_3_ flake (Figure 2b).

Taking into account the energy values for the electron affinity and bandgap for monolayer MoS_2_ reported in the literature,^54^ ∼4.3 and ∼1.89 eV, respectively, and considering a work function of ∼4.8 eV for exfoliated 1L MoS_2_ measured in ambient conditions,^55^ a diagram of the energy band alignment for the 1L MoS_2_/FePS_3_ heterostructure has been built (Figure 2b). The justification for using work function values obtained in vacuum and in air for FePS_3_ and 1L MoS_2_, respectively, falls on the fact that the MoS_2_ monolayer may act as an encapsulating material for the area of FePS_3_ on which it is deposited. We indeed observe that the PL is quenched if the samples are not prepared under a controlled atmosphere, whereas the PL enhancement of heterostructures prepared in a controlled environment can be observed even after months of preparation.

In Figure 2c, the valence band maximum (VBM) of FePS_3_ is located above the VBM of 1L MoS_2_, whereas the conduction band minimum (CBM) of 1L MoS_2_ is below the CBM of FePS_3_. Therefore, for the van der Waals heterojunction, the VBM and CBM are localized on FePS_3_ and MoS_2_, respectively, confirming a type II heterointerface.The exact location of the bands for FePS_3_ has an estimated error of about ±0.2 eV due to the uncertainty in the determination of the UPS slope and the lack of an exact determination of dopant and free carrier densities. There is also a similar range of variation in the reported energy positions for the MoS_2_ levels. Even taking those uncertainties into account, the qualitative description of a type II band alignment holds. In this scenario, the observed depletion of the MoS_2_ layer must arise from the transfer of free electrons from the conduction band of 1L MoS_2_ to the available states in the FePS_3_ valence band. Moreover, we observe a small increase in the exciton lifetime (see Supporting Information Section S12) associated with the increase of its relative spectral weight in agreement with other works.^33,38,39^ Furthermore, the fact that photoluminescence quenches in heterostructures prepared under a normal atmosphere (see Supporting Information Section S11) indicates that mechanisms requiring atomic proximity are responsible for the observed PL changes. This allows us to discard other leading mechanisms such as long-range energy transfer in our samples.

In the absence of dopants, charge transfer would be very limited by the unfavorable conditions provided by a pristine heterostructure in which both materials end up in sulfur atoms. To demonstrate this, we have carried out Hubbard-corrected DFT calculations (see computational details in Supporting Information Section S9) followed by a charge transfer Bader analysis. For simplicity, we have focused on a system formed by a bilayer MoS_2_/FePS_3_ (Figures 2c and S9). The Bader analysis, in agreement with the charge transfer analysis obtained from the *ab initio* calculations, indicates that only a small portion of the charge is transferred between the two stacked materials (see Figure 2c and details in Table S3) and that the charge redistribution occurs only inside each material. We conclude that for the case where FePS_3_ and MoS_2_ are intrinsic semiconductors, charge transfer between both materials is negligible. Then, we provide an estimation of the band alignment of bulk FePS_3_ and single-layer MoS_2_ using an ML slab model (see Computational Details in Methods). Work function values obtained from DFT calculations for defect-free intrinsic crystals of MoS_2_ and FePS_3_ yield a type I band alignment, regardless of the thickness of FePS_3_ (see Supporting Information Section S9, Figures S10 and S11). To provide a more realistic picture, which contemplates the existence of dopants, we calculate the electronic structure of MoS_2_ in the presence of S vacancies using a 4 × 4 × 1 supercell (see Section S10, Figure S12). This picture results in a type II band alignment between FePS_3_ and vacant MoS_2_ (see Section S10, Figure S13) and provides a closer description of the experimental results, suggesting, due to the chemical similarity, the presence of sulfur vacancies also in FePS_3._ These can adsorb oxygen atoms and induce oxidation of Fe^2+^ to Fe^3+^ that facilitates charge transfer at the interface.

We conclude that the strong electron acceptor character of naturally doped FePS_3_ combined with the natural electron doping of MoS_2_ are the key features, together with a favorable band alignment, that facilitate the observed charge transfer. Charge conservation requires that electron depletion in MoS_2_ is accompanied by a similar amount of hole depletion at FePS_3_. This creates a built-in potential across the junction that, in our case, acts as an energy barrier preventing the nonradiative recombination of photogenerated carriers and, thus, preserving the excitons and their photoluminescent recombination in MoS_2_. Furthermore, the fact that the VBM of FePS_3_ and the CBM of MoS_2_ have different momentum (see band structure calculations in Section S9), prevents the formation of interlayer excitons.

While charge transfer in the MoS_2_ is limited to a single layer, in the case of FePS_3_, hole depletion can extend over several layers of the material. Indeed, we find that the thickness of FePS_3_ flakes limits the charge transfer. For FePS_3_ flakes with thicknesses above 100 nm, we observe a PL enhancement from two to four times larger than in the control sample (see Section S5, Figure S6), and depletion of MoS_2_ carriers larger than 95%. These are unusually high values, both for enhancement and depletion, in the case of van der Waals heterostructures. This is illustrated by comparing the emission of several samples with different FePS_3_ thicknesses which reveals a clear dependence on the estimated amount of charge transferred between MoS_2_ and FePS_3_ (Figure 2d). Roughly speaking, we can attribute the thickness dependence to a reduced number of acceptors available in the *p*-doped material compared to thicker FePS_3_. Also, while depletion at MoS_2_ must necessarily occur at the single layer, the interface equilibrium at the FePS_3_ side can result in an extended depletion layer, which can be of interest for photovoltaic or photodetection purposes. The reported photogating effects in FePS_3_^21^ could also play a role in the dynamic enhancement of the MoS_2_ depletion upon illumination.

**Figure 3:**
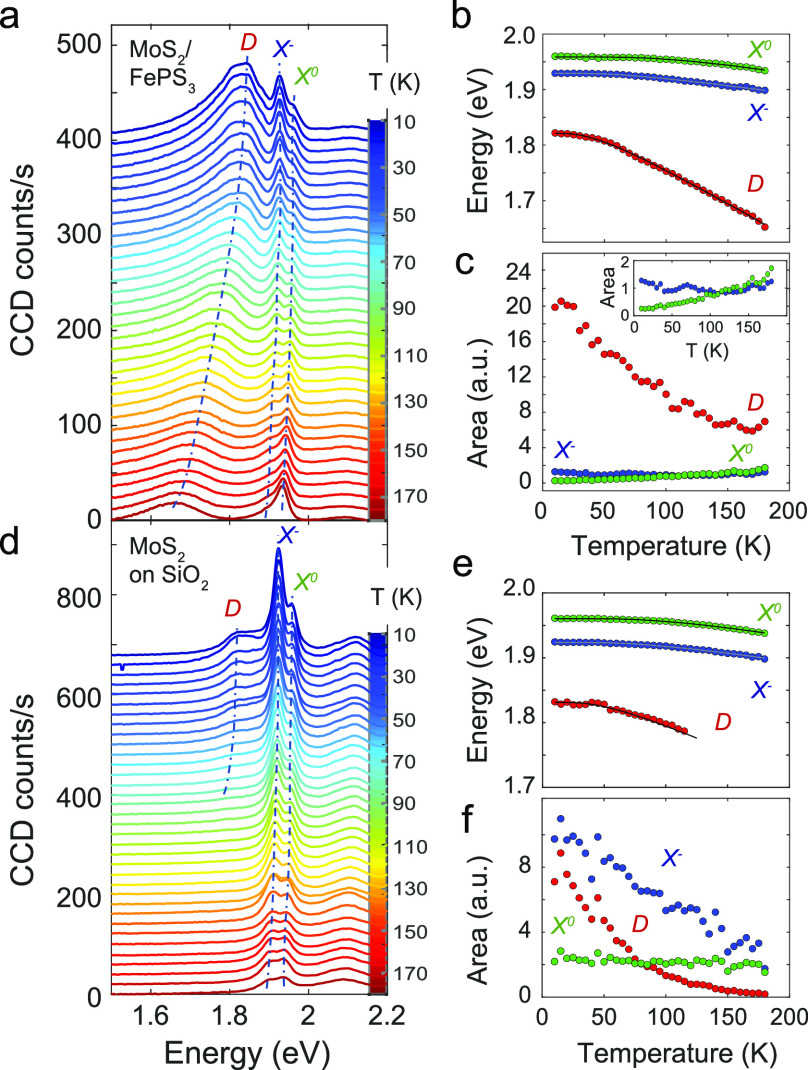
(a–c) Temperature evolution of photoluminescence within the range of 10–180 K in steps of 5 K in the heterostructure sample (a) PL spectra. (b) Peak energy positions extracted from a fit of the data to a multipeak model (see Supporting Information Section S13) as a function of temperature. The solid line represents the fit to a standard semiconductor model. (c) Peak areas. (d–f) Photoluminescence as a function of temperature in the control sample. (d) PL spectra. (e) Peak energy positions. (f) Peak areas.

To obtain more comprehensive details on the effects of charge transfer from the PL of 1L MoS_2_/FePS_3_ van der Waals heterostructure, temperature-dependent measurements have been carried out from 180 to 10 K (Figure 3a) in one of our heterostructures and contrasted with the low-temperature PL emission from the control sample (Figure 3d). In our analysis, we focus on the three more prominent PL peaks, which are labeled as *D*, *X*^–^, and *X*^0^ in Figure 3a,d, and obviate the peak related to exciton *B* (located at ∼2.1 eV) (see fit details in Supporting Information Section S13).

Clearly, peak *D* is evident in the heterostructure in the full range of temperatures, whereas in the control sample, it starts to be more appreciable only below 80 K. This peak, moving between 1.6 and 1.8 eV depending on temperature, has been observed previously in the PL emission of single-layer MoS_2_ and has been attributed to the radiative recombination of excitons bounded to intragap defects formed from sulfur vacancies^57,58^^.^

We observe that for both samples, control and heterostructure, the positions of the three peaks, *D*, *X*^–^, and *X*^0^, are all blue shifted as temperature diminishes (Figure 3b,e). This is attributed to a decreased electron-phonon interaction as well as to small changes in the bonding length.^59^ To quantify the blue shifting of the PL emission in the heterostructure and control samples when decreasing temperature, a standard semiconducting bandgap model has been used (see ref (60) and Section S14).

The parameters obtained from fitting the evolution of peak energy positions with temperature to the model are summarized in Table S4 and are consistent with the previous works^36^ for the case of the two excitonic peaks *X*^–^ and *X*^0^. From these values, the trion binding energies for the heterostructure and control samples are similar, being ∼30 and ∼36 meV, respectively. We attribute the small difference in binding energies between the samples to the different local dielectric screening of the Coulomb interaction in the MoS_2_ monolayers.^61^ On the other hand, the larger energy shift of peak *D* with varying temperature is also manifested through a higher electron–phonon coupling strength in contrast with the one obtained for the two excitonic peaks, *X*^–^ and *X*^0^, in both samples (see fitted values for parameter *S* in Table S4).

There is also a temperature-dependent change in the relative spectral weight between *X*^–^ and *X*^0^ emission peaks (Figure 3c). This gradual change of trion-exciton contribution is also observed in the control sample (Figure 3f). This observation has been previously attributed to electrons escaping their trion-bound state owing to thermal fluctuations.^62^

The spectral weight of the PL peak associated with defect-bound excitons increases significantly with decreasing temperature. This behavior has been observed in different single-layer TMDs,^4,63,64^ follows an Arrhenius trend with activation energies in the order of tens of meV (see Section S15), and has been attributed to an increase of nonradiative recombination processes with temperature^4,63^ or to a possible charged nature of bound excitons.^64^

More interestingly, the remarkable increase of the defect peak in the heterostructure corroborates the abovementioned scenario of electron transfer. In the work presented by Greben *et al*.,^63^ a law of mass action is introduced to describe the equilibrium between the density of free excitons and exciton bound by defects: *X*^0^ + *d* → *D*. The rate between those densities is, in this case, governed by the density of unoccupied dopant levels in MoS_2_
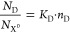
3where *N*_D_ and *N*_*X*^0^_ are the density of defect-related excitons and trions, respectively, while *K*_D_ and *n*_D_ are the rate constants for defect-bound excitons and the concentration of unoccupied in-gap defect levels, respectively.

Similarly, the ratio between free carrier density in the heterostructure and control samples can be attributed to the proportion in spectral weight between defect and exciton emission peaks, which is directly related to their respective populations
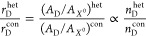
4

This analysis shows that in the heterostructure and below 100 K, there are 20–25 times more unoccupied defects than in the control sample (Figure 3c). This is compatible with the electron depletion of MoS_2_, which in ref (63) is achieved by the application of an external electric field and is caused here by the acceptor character of FePS_3_. This was already qualitatively observable by the fact that at 180 K, a defect peak is present in the heterostructure but not in the control sample. Because of charge transfer, the photoluminescence of MoS_2_ in the heterostructure resembles that of a semiconductor with a lower degree of doping than in the case of the control sample (Figure 3a–c).

## Conclusions

In summary, our study corroborates an efficient electron transfer from the n-doped MoS_2_ monolayer to the p-doped multilayer FePS_3_ flake by combining optical spectroscopy, UPS, *ab initio* calculations, low-temperature transport, and PL measurements. The charge transfer signatures obtained in the 2D heterostructure *via* PL measurements at room temperature are comparable to the ones achieved *via* chemical functionalization, where preservation or enhancement of the PL efficiency is accomplished. We attribute the charge transfer and the preservation of PL to the very favorable band alignment of the heterostructure. Our results suggest that the light emission properties of single-layer, n-type TMDs can be improved not only in some type I semiconductor heterostructures, but also in type II arrangements with indirect, smaller gap p-type semiconductors.

The enhancement and narrowing of the PL emission could inspire the design of future highly efficient light-emitting diodes based on band alignment engineering of heterostructures composed of atomically thin MoS_2_. Through a careful analysis of several heterostructures, we are able to track the dependence of the number of electrons removed from single-layer MoS_2_ as a function of the thickness of the FePS_3_ underneath. Thus, charge transfer and, consequently, PL can be easily tuned by a proper thickness selection of FePS_3_, enabling convenient control of optical and electrical properties of atomically thin MoS_2_. The singular PL tunability of the system invites us to continue exploring this 2D heterostructure as an optoelectronic material, where a meticulous study of the leading mechanisms between electron–hole recombinations and/or dissociations can have an impact on the efficiency of photodetectors, photovoltaic cells, light-emitting diodes, or electroluminescent junctions based on 2D materials.

## Methods

### Fabrication of Vertical Single-Layer MoS_2_/MultiLayer FePS_3_ Heterostructures

Commercially available MoS_2_ (SPI Supplies) and lab-grown FePS_3_*via* chemical vapor transport^65^ were mechanically exfoliated onto transparent polydimethylsiloxane (PDMS) substrates. Optical microscopy, micro-reflectance, and Raman spectroscopies enabled us to identify the thickness of FePS_3_ and MoS_2_ flakes (see Supporting Information Sections S1 and S2). After identification, the selected flakes were deposited onto a 300 nm-thick SiO_2_/Si substrate *via* a deterministic, dry transfer method^66^ to form vertically stacked heterostructures. The exfoliation of FePS_3_ flakes and the heterostructure fabrication was performed in an inert Argon atmosphere.

### Photoluminescence Characterization

PL measurements at room temperature were performed using a commercial Raman microscope (Jasco NRS-5100) using an excitation line of 532 nm, with a laser spot of ∼1.5 μm diameter and a total power of 60 μW. Low-temperature micro-PL measurements were carried out using a diffraction-limited fiber in a confocal setup inserted into a pulse-tube-based closed-cycle Helium cryostat (attoDRY 2100, Attocube). A 532 nm solid-state laser was used with an irradiated laser power of approximately 100 μW at the sample.

### Ultraviolet Photoelectron Spectroscopy

He I (ℏω = 21.22 eV) UPS spectra were taken on bulk FePS_3_ crystals. Samples were exfoliated while already mounted in the experiment chamber in order to reduce the air exposure of the surface down to a few seconds. A bias voltage of −10 V was applied to the sample in order to differentiate the secondary electron cut-off.

### Computational Details

The electronic structure of MoS_2_/FePS_3_ heterostructure was calculated using the first-principles plane-wave DFT + U approach as implemented in the Quantum ESPRESSO package,^67^ using a Hubbard U (on-site Coulomb repulsion) of 2.2 eV, as reported in ref (21) (see also Supporting Information Section S9 for more details). All chemical structures were fully optimized using the Broyden–Fletcher–Goldfarb–Shanno (BFGS) algorithm^68^ until the forces on each atom were smaller than 1 × 10^–3^ Ry/au and the energy difference between two consecutive relaxation steps was less than 1 × 10^–4^ Ry. The Brillouin zone was sampled at least by a fine Γ-centered 4 × 4 × 1 *k*-point Monkhorst–Pack mesh^69^ for all monolayer calculations choosing a well converged third *k* point according to the length of slabs. The heterostructure was set up by a 2 × 2 hexagonal supercell of single-layer FePS_3_, keeping the fully optimized lattice parameters from the bulk, combined with a 4 × 4 MoS_2_ supercell, assuming a 7.19% mismatch for the MoS_2_. The stacking was based on previous works with analogous materials.^70^ An extended mesh of 8 × 8 × 2 *k*-points was necessary to determine the charge transfer between the layers and converge the charges during the Bader analysis. The work function was determined for MoS_2_ and FePS_3_ monolayers and bulk FePS_3_, which was simulated with slabs formed by 4 and 6 layers, being already converged in the 4-layers slab calculation. To evaluate the presence of defects in the work function of MoS_2_, we built up a 4 × 4 × 1 supercell to isolate a S vacancy.

## Supporting Information Available

Raman spectroscopy; thickness estimation of FePS_3_; quantitative analysis of the fitting parameters obtained from Figure 1; mass action law; PL spectroscopy of heterostructures with different thicknesses and their corresponding charge transfer; valence band UPS spectrum of bulk FePS_3_; Kelvin probe force microscopy; thermal activation energy in FePS_3_; computational methods for pristine MoS_2_/FePS_3_; theoretical analysis of sulfur vacancy in MoS_2_; samples prepared in air; exciton lifetime characterization; Lorentzian peak fittings of PL spectra at low temperature; semiconductor bandgap model; data analysis of defects peak at low temperature; and activation energies of sulfur vacancies (PDF)

## Abbreviations

2D: two-dimensional
TMD: transition metal dichalcogenide
MPX_3_: transition metal chalcogenophosphate
PL: photoluminescence
1L: one layer
ML: multilayer
UPS: ultraviolet photoelectron spectroscopy
VBM: valence band maximum
CBM: conduction band minimum
SI: Supporting Information

## References

[ref1] Novoselov K. S.; Geim A. K.; Morozov S. V.; Jiang D. E.; Zhang Y.; Dubonos S. V.; Grigorieva I. V.; Firsov A. A. Electric Field Effect in Atomically Thin Carbon Films. Science 2004, 306, 666–669. 10.1126/science.1102896. 15499015

[ref2] Splendiani A.; Sun L.; Zhang Y.; Li T.; Kim J.; Chim C. Y.; Galli G.; Wang F. Emerging Photoluminescence in Monolayer MoS2. Nano Lett. 2010, 10, 1271–1275. 10.1021/nl903868w. 20229981

[ref3] Yin Z.; Li H.; Li H.; Jiang L.; Shi Y.; Sun Y.; Lu G.; Zhang Q.; Chen X.; Zhang H. Single-Layer MoS2 Phototransistors. ACS Nano 2012, 6, 74–80. 10.1021/nn2024557. 22165908

[ref4] Korn T.; Heydrich S.; Hirmer M.; Schmutzler J.; Schüller C. Low-Temperature Photocarrier Dynamics in Monolayer MoS2. Appl. Phys. Lett. 2011, 99, 102109 10.1063/1.3636402.

[ref5] Zeng H.; Dai J.; Yao W.; Xiao D.; Cui X. Valley Polarization in MoS2 Monolayers by Optical Pumping. Nat. Nanotechnol. 2012, 7, 490–493. 10.1038/nnano.2012.95. 22706701

[ref6] Wang Q. H.; Kalantar-Zadeh K.; Kis A.; Coleman J. N.; Strano M. S. Electronics and Optoelectronics of Two-Dimensional Transition Metal Dichalcogenides. Nat. Nanotechnol. 2012, 7, 699–712. 10.1038/nnano.2012.193. 23132225

[ref7] Kumar A.; Ahluwalia P. K. A First Principle Comparative Study of Electronic and Optical Properties of 1H–MoS2 and 2H–MoS2. Mater. Chem. Phys. 2012, 135, 755–761. 10.1016/j.matchemphys.2012.05.055.

[ref8] Furchi M. M.; Pospischil A.; Libisch F.; Burgdörfer J.; Mueller T. Photovoltaic Effect in an Electrically Tunable Van Der Waals Heterojunction. Nano Lett. 2014, 14, 4785–4791. 10.1021/nl501962c. 25057817 PMC4138224

[ref9] Yang W.; Kawai H.; Bosman M.; Tang B.; Chai J.; Tay W.; Yang J.; Seng H. S.; Zhu H.; Gong H.; Liu H.; Goh K. E. J.; Wang S.; Chi D. Interlayer Interactions in 2D WS2/MoS2 Heterostructures Monolithically Grown by in Situ Physical Vapor Deposition. Nanoscale 2018, 10, 22927–22936. 10.1039/c8nr07498d. 30499578

[ref10] Yuan J.; Najmaei S.; Zhang Z.; Zhang J.; Lei S.; Ajayan P. M.; Yakobson B. I.; Lou J. Photoluminescence Quenching and Charge Transfer in Artificial Heterostacks of Monolayer Transition Metal Dichalcogenides and Few-Layer Black Phosphorus. ACS Nano 2015, 9, 555–563. 10.1021/nn505809d. 25569715

[ref11] Huang B.; McGuire M. A.; May A. F.; Xiao D.; Jarillo-Herrero P.; Xu X. Emergent Phenomena and Proximity Effects in Two-Dimensional Magnets and Heterostructures. Nat. Mater. 2020, 19, 1276–1289. 10.1038/s41563-020-0791-8. 32948831

[ref12] Lee J. U.; Lee S.; Ryoo J. H.; Kang S.; Kim T. Y.; Kim P.; Park C.; Park J.; Cheong H. Ising-Type Magnetic Ordering in Atomically Thin FePS3. Nano Lett. 2016, 16, 7433–7438. 10.1021/acs.nanolett.6b03052. 27960508

[ref13] Wang X.; Du K.; Fredrik Liu Y. Y. F.; Hu P.; Zhang J.; Zhang Q.; Owen M. H. S.; Lu X.; Gan C. K.; Sengupta P.; Kloc C.; Xiong Q. Raman spectroscopy of atomically thin two-dimensional magnetic iron phosphorus trisulfide (FePS 3) crystals. 2D Materials 2016, 3, 031009 10.1088/2053-1583/3/3/031009.

[ref14] Liu Q.; Wang L.; Fu Y.; Zhang X.; Huang L.; Su H.; Lin J.; Chen X.; Yu D.; Cui X.; Mei J. W.; Dai J. F. Magnetic order in XY-type antiferromagnetic monolayer CoPS3 revealed by Raman spectroscopy. Phys. Rev. B 2021, 103, 235411 10.1103/physrevb.103.235411.

[ref15] Sun Y. J.; Tan Q. H.; Liu X. L.; Gao Y. F.; Zhang J. Probing the Magnetic Ordering of Antiferromagnetic MnPS3 by Raman Spectroscopy. J. Phys. Chem. Lett. 2019, 10, 3087–3093. 10.1021/acs.jpclett.9b00758. 31088058

[ref16] Mai T. T.; Garrity K. F.; McCreary A.; Argo J.; Simpson J. R.; Doan-Nguyen V.; Aguilar R. V.; Walker A. R. H. Magnon-Phonon Hybridization in 2D Antiferromagnet MnPSe3. Sci. Adv. 2021, 7, eabj3106 10.1126/sciadv.abj3106. 34714675 PMC8555890

[ref17] Kang S.; Kim K.; Kim B. H.; Kim J.; Sim K. I.; Lee J. U.; Lee S.; Park K.; Yun S.; Kim T.; Nag A.; Walters A.; Garcia-Fernandez M.; Li J.; Chapon L.; Zhou K. J.; Son Y. W.; Kim J. H.; Cheong H.; Park J. G. Coherent Many-Body Exciton in Van Der Waals Antiferromagnet NiPS3. Nature 2020, 583, 785–789. 10.1038/s41586-020-2520-5. 32690938

[ref18] Hwangbo K.; Zhang Q.; Jiang Q.; Wang Y.; Fonseca J.; Wang C.; Diederich G. M.; Gamelin D. R.; Xiao D.; Chu J. H.; Yao W.; Xu X. Highly Anisotropic Excitons and Multiple Phonon Bound States in a Van Der Waals Antiferromagnetic Insulator. Nat. Nanotechnol. 2021, 16, 655–660. 10.1038/s41565-021-00873-9. 33707746

[ref19] Zhang Q.; Hwangbo K.; Wang C.; Jiang Q.; Chu J. H.; Wen H.; Xiao D.; Xu X. Observation of Giant Optical Linear Dichroism in a Zigzag Antiferromagnet FePS3. Nano Lett. 2021, 21, 6938–6945. 10.1021/acs.nanolett.1c02188. 34428905

[ref20] Gao Y.; Lei S.; Kang T.; Fei L.; Mak C. L.; Yuan J.; Zhang M.; Li S.; Bao Q.; Zeng Z.; Wang Z.; Gu H.; Zhang K. Bias-Switchable Negative and Positive Photoconductivity in 2D FePS3 Ultraviolet Photodetectors. Nanotechnology 2018, 29, 244001 10.1088/1361-6528/aab9d2. 29582784

[ref21] Ramos M.; Carrascoso F.; Frisenda R.; Gant P.; Mañas-Valero S.; Esteras D. L.; Baldoví J. J.; Coronado E.; Castellanos-Gomez A.; Calvo M. R. Ultra-Broad Spectral Photo-Response in FePS3 Air-Stable Devices. npj 2D Mater. Appl. 2021, 5, 19 10.1038/s41699-021-00199-z.

[ref22] Kumar R.; Jenjeti R. N.; Austeria M. P.; Sampath S. Bulk and Few-Layer MnPS3: a New Candidate for Field Effect Transistors and UV Photodetectors. J. Mater. Chem. C 2019, 7, 324–329. 10.1039/c8tc05011b.

[ref23] Ou Z.; Wang T.; Tang J.; Zong X.; Wang W.; Guo Q.; Xu Y.; Zhu C.; Wang L.; Huang W.; Xu H. Enabling and Controlling Negative Photoconductance of FePS 3 Nanosheets by Hot Carrier Trapping. Adv. Opt. Mater. 2020, 8, 2000201 10.1002/adom.202000201.

[ref24] Chu J.; Wang F.; Yin L.; Lei L.; Yan C.; Wang F.; Wen Y.; Wang Z.; Jiang C.; Feng L.; Xiong J.; Li Y.; He J. High-Performance Ultraviolet Photodetector Based on a Few-Layered 2D NiPS3 Nanosheet. Adv. Funct. Mater. 2017, 27, 1701342 10.1002/adfm.201701342.

[ref25] Jenjeti R. N.; Kumar R.; Austeria M. P.; Sampath S. Field Effect Transistor Based on Layered NiPS 3. Sci. Rep. 2018, 8, 8586 10.1038/s41598-018-26522-1. 29872067 PMC5988702

[ref26] Xu T.; Luo M.; Shen N.; Yu Y.; Wang Z.; Cui Z.; Qin J.; Liang F.; Chen Y.; Zhou Y.; Zhong F.; Peng M.; Zubair M.; Li N.; Miao J.; Lu W.; Yu C.; Hu W. Ternary 2D Layered Material FePSe 3 and Near-Infrared Photodetector. Adv. Electron. Mater. 2021, 7, 2100207 10.1002/aelm.202100207.

[ref27] Xing W.; Qiu L.; Wang X.; Yao Y.; Ma Y.; Cai R.; Jia S.; Xie X. C.; Han W. Magnon Transport in Quasi-Two-Dimensional Van Der Waals Antiferromagnets. Phys. Rev. X 2019, 9, 011026 10.1103/physrevx.9.011026.

[ref28] McCreary A.; Simpson J. R.; Mai T. T.; McMichael R. D.; Douglas J. E.; Butch N.; Dennis C.; Aguilar R. V.; Walker A. R. H. Quasi-Two-Dimensional Magnon Identification in Antiferromagnetic FePS3 via Magneto-Raman Spectroscopy. Phys. Rev. B 2020, 101, 064416 10.1103/physrevb.101.064416. PMC11015466 38616972

[ref29] Ghosh A.; Palit M.; Maity S.; Dwij V.; Rana S.; Datta S. Spin-Phonon Coupling and Magnon Scattering in Few-Layer Antiferromagnetic FePS3. Phys. Rev. B 2021, 103, 064431 10.1103/physrevb.103.064431.

[ref30] Zhang X. X.; Jiang S.; Lee J.; Lee C.; Mak K. F.; Shan J. Spin Dynamics Slowdown Near the Antiferromagnetic Critical Point in Atomically Thin FePS3. Nano Lett. 2021, 21, 5045–5052. 10.1021/acs.nanolett.1c00870. 34106709

[ref31] Liu S.; Granados Del Águila A. G.; Bhowmick D.; Gan C. K.; Thu Ha Do T. T. H.; Prosnikov M. A.; Sedmidubský D.; Sofer Z.; Christianen P. C. M.; Sengupta P.; Xiong Q. Direct Observation of Magnon-Phonon Strong Coupling in Two-Dimensional Antiferromagnet at High Magnetic Fields. Phys. Rev. Lett. 2021, 127, 097401 10.1103/PhysRevLett.127.097401. 34506201

[ref32] Boström E. V.; Parvini T. S.; McIver J. W.; Rubio A.; Kusminskiy S. V.; Sentef M. A. All-Optical Generation of Antiferromagnetic Magnon Currents via the Magnon Circular Photogalvanic Effect. Phys. Rev. B 2021, 104, L100404 10.1103/physrevb.104.l100404.

[ref33] Amani M.; Lien D. H.; Kiriya D.; Xiao J.; Azcatl A.; Noh J.; Madhvapathy S. R.; Addou R.; Kc S.; Dubey M.; Cho K.; Wallace R. M.; Lee S. C.; He J. H.; Ager J. W.; Zhang X.; Yablonovitch E.; Javey A. Near-Unity Photoluminescence Quantum Yield in MoS2. Science 2015, 350, 1065–1068. 10.1126/science.aad2114. 26612948

[ref34] Bertolazzi S.; Gobbi M.; Zhao Y.; Backes C.; Samorì P. Molecular Chemistry Approaches for Tuning the Properties of Two-Dimensional Transition Metal Dichalcogenides. Chem. Soc. Rev. 2018, 47, 6845–6888. 10.1039/c8cs00169c. 30043037

[ref35] Mouri S.; Miyauchi Y.; Matsuda K. Tunable Photoluminescence of Monolayer MoS2 via Chemical Doping. Nano Lett. 2013, 13, 5944–5948. 10.1021/nl403036h. 24215567

[ref36] Wang Y.; Slassi A.; Stoeckel M. A.; Bertolazzi S.; Cornil J.; Beljonne D.; Samorì P. Doping of Monolayer Transition-Metal Dichalcogenides via Physisorption of Aromatic Solvent Molecules. J. Phys. Chem. Lett. 2019, 10, 540–547. 10.1021/acs.jpclett.8b03697. 30649889

[ref37] Duan J.; Chava P.; Ghorbani-Asl M.; Erb D.; Hu L.; Krasheninnikov A. V.; Schneider H.; Rebohle L.; Erbe A.; Helm M.; Zeng Y. J.; Zhou S.; Prucnal S. Enhanced Trion Emission in Monolayer MoSe 2 by Constructing a Type-I Van Der Waals Heterostructure. Adv. Funct. Mater. 2021, 31, 2104960 10.1002/adfm.202104960.

[ref38] Zheng W.; Zheng B.; Yan C.; Liu Y.; Sun X.; Qi Z.; Yang T.; Jiang Y.; Huang W.; Fan P.; Jiang F.; Ji W.; Wang X.; Pan A. Direct Vapor Growth of 2D Vertical Heterostructures with Tunable Band Alignments and Interfacial Charge Transfer Behaviors. Adv. Sci. 2019, 6, 1802204 10.1002/advs.201802204. PMC6446596 30989032

[ref39] Zhang D.; Liu Y.; He M.; Zhang A.; Chen S.; Tong Q.; Huang L.; Zhou Z.; Zheng W.; Chen M.; Braun K.; Meixner A. J.; Wang X.; Pan A. Room Temperature Near Unity Spin Polarization in 2D Van Der Waals Heterostructures. Nat. Commun. 2020, 11, 4442 10.1038/s41467-020-18307-w. 32895376 PMC7477097

[ref40] Gong Y.; Lin J.; Wang X.; Shi G.; Lei S.; Lin Z.; Zou X.; Ye G.; Vajtai R.; Yakobson B. I.; Terrones H.; Terrones M.; Tay B. K.; Lou J.; Pantelides S. T.; Liu Z.; Zhou W.; Ajayan P. M. Vertical and In-Plane Heterostructures from WS2/MoS2 Monolayers. Nat. Mater. 2014, 13, 1135–1142. 10.1038/nmat4091. 25262094

[ref41] Yu Y.; Hu S.; Su L.; Huang L.; Liu Y.; Jin Z.; Purezky A. A.; Geohegan D. B.; Kim K. W.; Zhang Y.; Cao L. Equally Efficient Interlayer Exciton Relaxation and Improved Absorption in Epitaxial and Nonepitaxial MoS2/WS2 Heterostructures. Nano Lett. 2015, 15, 486–491. 10.1021/nl5038177. 25469768

[ref42] Xiao J.; Liu J.; Sun K.; Zhao Y.; Shao Z.; Liu X.; Yuan Y.; Li Y.; Xie H.; Song F.; Gao Y.; Huang H. PbI2-MoS2 Heterojunction: van der Waals Epitaxial Growth and Energy Band Alignment. J. Phys. Chem. Lett. 2019, 10, 4203–4208. 10.1021/acs.jpclett.9b01665. 31291727

[ref43] Boix-Constant C.; García-López V.; Navarro-Moratalla E.; Clemente-León M.; Zafra J. L.; Casado J.; Guinea F.; Mañas-Valero S.; Coronado E. Strain Switching in Van Der Waals Heterostructures Triggered by a Spin-Crossover Metal Organic Framework. Adv. Mater. 2022, 34, 2110027 10.1002/adma.202110027. 35032055

[ref44] Torres-Cavanillas R.; Morant-Giner M.; Escorcia-Ariza G.; Dugay J.; Canet-Ferrer J.; Tatay S.; Cardona-Serra S.; Giménez-Marqués M.; Galbiati M.; Forment-Aliaga E.; Coronado E. Spin-Crossover Nanoparticles Anchored on MoS2 Layers for Heterostructures with Tunable Strain Driven by Thermal or Light-Induced Spin Switching. Nat. Chem. 2021, 13, 1101–1109. 10.1038/s41557-021-00795-y. 34621077

[ref45] Ryu Y. K.; Carrascoso F.; López-Nebreda R.; Agraït N.; Frisenda R.; Castellanos-Gomez A. Microheater Actuators as a Versatile Platform for Strain Engineering in 2D Materials. Nano Lett. 2020, 20, 5339–5345. 10.1021/acs.nanolett.0c01706. 32491864

[ref46] Chaves A.; Azadani J. G.; Alsalman H.; Da Costa D. R.; Frisenda R.; Chaves A. J.; Song S. H.; Kim Y. D.; He D.; Zhou J.; Castellanos-Gomez A.; Peeters F. M.; Liu Z.; Hinkle C. L.; Oh S. H.; Ye P. D.; Koester S. J.; Lee Y. H.; Avouris P.; Wang X.; Low T. Bandgap Engineering of Two-Dimensional Semiconductor Materials. npj 2D Mater. Appl. 2020, 4, 29 10.1038/s41699-020-00162-4.

[ref47] Zhang X.; Nan H.; Xiao S.; Wan X.; Ni Z.; Gu X.; Ostrikov K. Shape-Uniform, High-Quality Monolayered MoS2 Crystals for Gate-Tunable Photoluminescence. ACS Appl. Mater. Interfaces 2017, 9, 42121–42130. 10.1021/acsami.7b14189. 29111648

[ref48] Mak K. F.; He K.; Lee C.; Lee G. H.; Hone J.; Heinz T. F.; Shan J. Tightly Bound Trions in Monolayer MoS2. Nat. Mater. 2013, 12, 207–211. 10.1038/nmat3505. 23202371

[ref49] Helander M. G.; Greiner M. T.; Wang Z. B.; Lu Z. H. Pitfalls in Measuring Work Function using Photoelectron Spectroscopy. Appl. Surf. Sci. 2010, 256, 2602–2605. 10.1016/j.apsusc.2009.11.002.

[ref50] Ozawa K. Ultraviolet Photoelectron Spectroscopy. Compendium of Surface and Interface Analysis; Springer: Singapore, 2018; pp 783–790.

[ref51] Duan J.; Chava P.; Ghorbani-Asl M.; Lu Y.; Erb D.; Hu L.; Echresh A.; Rebohle L.; Erbe A.; Krasheninnikov V.; Helm M.; Zeng Y. J.; Zhou S.; Prucnal S. Self-Driven Broadband Photodetectors Based on MoSe2/FePS3 van der Waals n-p Type-II Heterostructures. ACS Appl. Mater. Interfaces 2022, 14, 11927–11936. 10.1021/acsami.1c24308. 35191687

[ref52] Budniak A. K.; Zelewski S. J.; Birowska M.; Woźniak T.; Bendikov T.; Kauffmann Y.; Amouyal Y.; Kudrawiec R.; Lifshitz E. Spectroscopy and Structural Investigation of Iron Phosphorus Trisulfide-FePS 3. Adv. Opt. Mater. 2022, 10, 2102489 10.1002/adom.202102489.

[ref53] Grasso V.; Neri F.; Patanè S.; Silipigni L.; Piacentini M. Conduction processes in the layered semiconductor compoundFePS3. Phys. Rev. B 1990, 42, 1690 10.1103/physrevb.42.1690. 9995599

[ref54] Frisenda R.; Molina-Mendoza A. J.; Mueller T.; Castellanos-Gomez A.; van der Zant H. S. Atomically thin p-n junctions based on two-dimensional materials. Chem. Soc. Rev. 2018, 47, 3339–3358. 10.1039/c7cs00880e. 29683464

[ref55] Tamulewicz M.; Kutrowska-Girzycka J.; Gajewski K.; Serafińczuk J.; Sierakowski A.; Jadczak J.; Bryja L.; Gotszalk T. P. Layer Number Dependence of the Work Function and Optical Properties of Single and Few Layers MoS2: Effect of substrate. Nanotechnology 2019, 30, 245708 10.1088/1361-6528/ab0caf. 30836333

[ref56] Kokalj A. XCrySDen--a new program for displaying crystalline structures and electron densities. J. Mol. Graph. Model. 1999, 17, 176–179. 10.1016/s1093-3263(99)00028-5. 10736774

[ref57] Mitterreiter E.; Schuler B.; Micevic A.; Hernangómez-Pérez D.; Barthelmi K.; Cochrane K. A.; Kiemle J.; Sigger F.; Klein J.; Wong E.; Barnard E. S.; Watanabe K.; Taniguchi T.; Lorke M.; Jahnke F.; Finley J. J.; Schwartzberg A. M.; Qiu D. Y.; Refaely-Abramson S.; Holleitner A. W.; Weber-Bargioni A.; Kastl C. The Role of Chalcogen Vacancies for Atomic Defect Emission in MoS2. Nat. Commun. 2021, 12, 3822 10.1038/s41467-021-24102-y. 34158488 PMC8219741

[ref58] Saigal N.; Ghosh S. Evidence for Two Distinct Defect Related Luminescence Features in Monolayer MoS2. Appl. Phys. Lett. 2016, 109, 122105 10.1063/1.4963133.

[ref59] Zhu S.; Zheng W. Temperature-Dependent Phonon Shifts in Van Der Waals Crystals. J. Phys. Chem. Lett. 2021, 12, 5261–5270. 10.1021/acs.jpclett.1c00947. 34060315

[ref60] O’donnell K. P.; Chen X. Temperature Dependence of Semiconductor Band Gaps. Appl. Phys. Lett. 1991, 58, 2924–2926. 10.1063/1.104723.

[ref61] Raja A.; Chaves A.; Yu J.; Arefe G.; Hill H. M.; Rigosi A. F.; Berkelbach T. C.; Nagler P.; Schüller C.; Korn T.; Nuckolls C.; Hone J.; Brus L. E.; Heinz T. F.; Reichman D. R.; Chernikov A. Coulomb Engineering of the Bandgap and Excitons in Two-Dimensional Materials. Nat. Commun. 2017, 8, 15251 10.1038/ncomms15251. 28469178 PMC5418602

[ref62] Ross J. S.; Wu S.; Yu H.; Ghimire N. J.; Jones A. M.; Aivazian G.; Yan J.; Mandrus D. G.; Xiao D.; Yao W.; Xu X. Electrical Control of Neutral and Charged Excitons in a Monolayer Semiconductor. Nat. Commun. 2013, 4, 1474 10.1038/ncomms2498. 23403575

[ref63] Greben K.; Arora S.; Harats M. G.; Bolotin K. I. Intrinsic and Extrinsic Defect-Related Excitons in TMDCs. Nano Lett. 2020, 20, 2544–2550. 10.1021/acs.nanolett.9b05323. 32191482

[ref64] Carozo V.; Wang Y.; Fujisawa K.; Carvalho B. R.; McCreary A.; Feng S.; Lin Z.; Zhou C.; Perea-López N.; Elías A. L.; Kabius B.; Crespi V. H.; Terrones M. Optical Identification of Sulfur Vacancies: Bound Excitons at the Edges of Monolayer Tungsten Disulfide. Sci. Adv. 2017, 3, e1602813 10.1126/sciadv.1602813. 28508048 PMC5409454

[ref65] Šiškins M.; Lee M.; Mañas-Valero S.; Coronado E.; Blanter Y. M.; van der Zant H. S.; Steeneken P. G. Magnetic and Electronic Phase Transitions Probed by Nanomechanical Resonators. Nat. Commun. 2020, 11, 2698 10.1038/s41467-020-16430-2. 32483113 PMC7264344

[ref66] Castellanos-Gomez A.; Buscema M.; Molenaar R.; Singh V.; Janssen L.; van der Zant H. S.; Steele G. A. Deterministic Transfer of Two-Dimensional Materials by All-Dry Viscoelastic Stamping. 2D Materials 2014, 1, 011002 10.1088/2053-1583/1/1/011002.

[ref67] Giannozzi P.; Baroni S.; Bonini N.; Calandra M.; Car R.; Cavazzoni C.; Ceresoli D.; Chiarotti G. L.; Cococcioni M.; Dabo I.; Dal Corso A.; de Gironcoli S.; Fabris S.; Fratesi G.; Gebauer R.; Gerstmann U.; Gougoussis C.; Kokalj A.; Lazzeri M.; Martin-Samos L.; Marzari N.; Mauri F.; Mazzarello R.; Paolini S.; Pasquarello A.; Paulatto L.; Sbraccia C.; Scandolo S.; Sclauzero G.; Seitsonen A. P.; Smogunov A.; Umari P.; Wentzcovitch R. M. QUANTUM ESPRESSO: A Modular and Open-Source Software Project for Quantum Simulations of Materials. J. Phys.: Condens. Matter 2009, 21, 395502 10.1088/0953-8984/21/39/395502. 21832390

[ref68] Head J. D.; Zerner M. C. A Broyden-Fletcher-Goldfarb-Shanno optimization procedure for molecular geometries. Chem. Phys. Lett. 1985, 122, 264–270. 10.1016/0009-2614(85)80574-1.

[ref69] Monkhorst H. J.; Pack J. D. Special Points for Brillouin-Zone Integrations. Phys. Rev. B 1976, 13, 5188 10.1103/physrevb.13.5188.

[ref70] Onga M.; Sugita Y.; Ideue T.; Nakagawa Y.; Suzuki R.; Motome Y.; Iwasa Y. Antiferromagnet-Semiconductor Van Der Waals Heterostructures: Interlayer Interplay of Exciton with Magnetic Ordering. Nano Lett. 2020, 20, 4625–4630. 10.1021/acs.nanolett.0c01493. 32407633

