# Supplementary material for: Photoluminescence Enhancement by Band Alignment Engineering in MoS2/FePS3 van der Waals Heterostructures
Source: ACS Appl Mater Interfaces. 2022 Jul 15;14(29):33482–90. doi: 10.1021/acsami.2c05464 (PMC9335528; doi:10.1021/acsami.2c05464)
Supplement: Supplementary file 1 — am2c05464_si_001.pdf [file am2c05464_si_001.pdf]

# Supporting Information

## Photoluminescence enhancement by band alignment engineering in $MoS_2$/$FePS_3$ van der Waals heterostructures

*Maria Ramos,*[*,†] Francisco Marques-Moros,[‡] Dorye L. Esteras,[‡] Samuel Mañas-Valero,[‡] Eudomar Henríquez-Guerra,[†] Marcos Gadea,[†] José J. Baldoví,[‡] Josep Canet-Ferrer,*[‡] Eugenio Coronado[‡] and M. Reyes Calvo*[†,§]*

†Departamento de Física Aplicada, Universidad de Alicante, Alicante, Spain

‡Instituto de Ciencia Molecular (ICMol), Universitat de València, Paterna, Spain

§Instituto Universitario de Materiales de Alicante (IUMA), Universidad de Alicante, Alicante, Spain

**Corresponding Authors**

*Maria Ramos – mramos@ua.es

*Josep Canet-Ferrer – jose.canet-ferrer@uv.es

*Reyes Calvo – reyes.calvo@ua.es

## Table of Contents

S1. Raman spectroscopy of single-layer $MoS_2$/ multi-layer $FePS_3$ van der Waals heterostacks

S2. Thickness estimation of $FePS_3$ flakes

S3. Quantitative analysis of the fitting parameters from Figure 1

S4. Estimation of the electron density in one-layer $MoS_2$

S5. Analysis of the Photoluminescence of 1L $MoS_2$/$FePS_3$ heterostructures with different $FePS_3$ thickness

S6. Valence band UPS spectrum of bulk $FePS_3$

S7. Kelvin probe force microscopy

S8. Thermal activation energy of a multi-layer $FePS_3$ flake

S9. Theoretical analysis of $MoS_2$/$FePS_3$

S10. Theoretical analysis of S vacancy in $MoS_2$

S11. Photoluminescence in heterostructures prepared in air

S12. Exciton lifetime characterization

S13. Four Lorentzian peak fitting of photoluminescence spectrum at low temperature

S14. Semiconductor bandgap model

S15. Activation energies of sulfur vacancies in heterostructure and control samples

## 1. Raman spectroscopy of single-layer $MoS_2$/ multi-layer $FePS_3$ van der Waals heterostacks

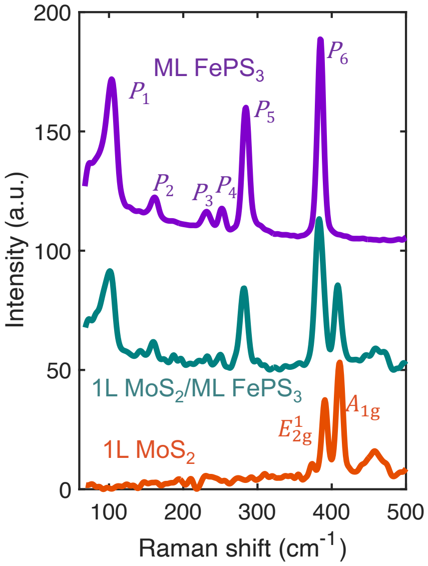


**Figure S1.** Raman spectra of single-layer $MoS_2$ (lower plot, 1L $MoS_2$), multilayer $FePS_3$ (upper plot, ML $FePS_3$) and their heterostructure (medium plot, 1L $MoS_2$/$FePS_3$).

Raman spectroscopy with a 532 nm excitation line has been employed to characterize the samples (Figure S1). The non-resonant Raman spectrum of a control sample (1L $MoS_2$ flake deposited onto 300-nm $SiO_2$/Si substrate) is dominated by two vibrational modes: $E^1_{2g}$, due to in-plane vibrations of two S atoms with respect to the Mo atom, and $A_{1g}$, due to the out-of-plane vibrations of S atoms in opposite directions. It is well-known that the frequency difference between these two peaks diminishes with lowering the number of material layers due to a reduced dielectric screening. The location of these two modes, 384 $cm^{-1}$ and 403 $cm^{-1}$, respectively, yields a difference in Raman shift of ~19 $cm^{-1}$ which is a distinctive signature of a single layer $MoS_2$ flake[1]. For a ML $FePS_3$ flake, six prominent vibrational modes are observed, which are in good agreement with previously reported works[2-5]. The Raman spectrum of 1L $MoS_2$/ML $FePS_3$ is formed by the sum of all the vibrational modes present in the Raman spectra of the individual materials composing the heterostructure.

## 2. Thickness estimation of $FePS_3$ flakes

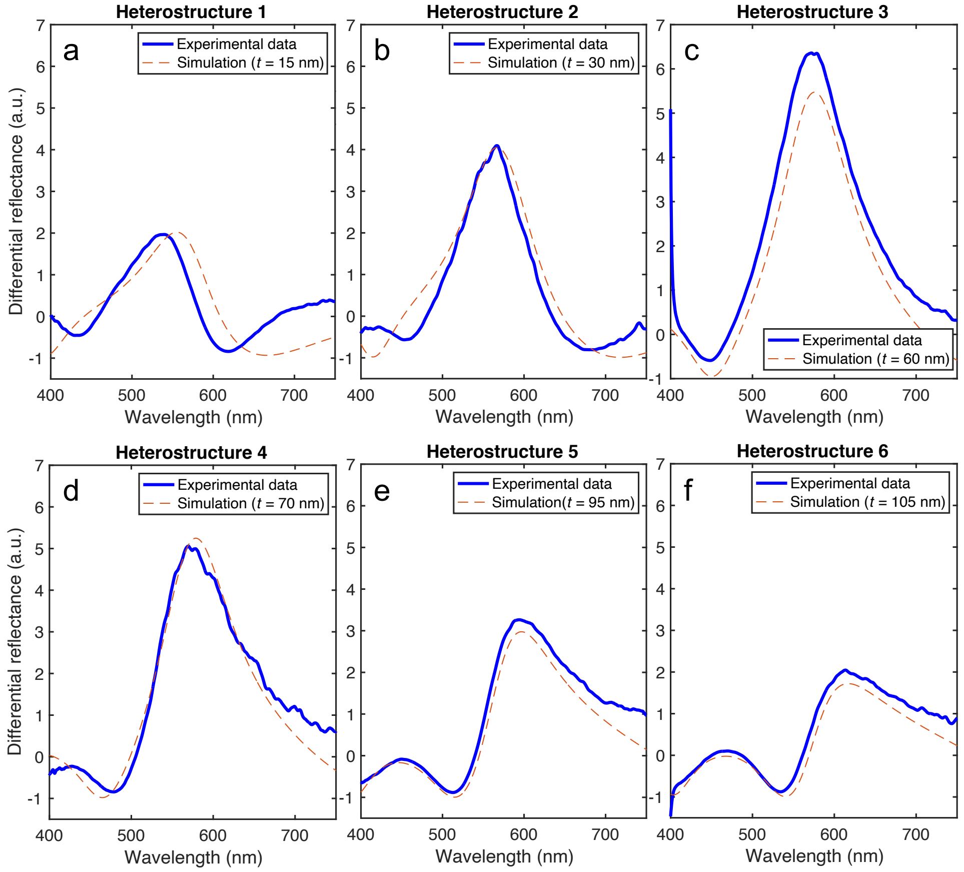


**Figure S2. (a)-(f)** Experimental and simulated differential reflectance spectra for $FePS_3$ flakes of several thicknesses.

Simulations of the differential reflectance spectra of $FePS_3$ exfoliated flakes onto $SiO_2$/Si substrates have been contrasted with their experimental counterparts to estimate the thickness of $FePS_3$ employed in all the fabricated $FePS_3$/1L-$MoS_2$ heterostructures. Specifically, the differential reflectance spectra shown in Figure S2 correspond to: $(R-R_0)/R_0$, being $R$ the reflectance of the $FePS_3$ flake and $R_0$ the reflectance of the $SiO_2$/Si substrate. The simulations are based on the transfer-matrix method for Fresnel equations, as in reference[6], accounting for a layer of Si with infinite thickness, a layer of $SiO_2$ with 295 nm thickness (previously adjusted under a similar optical reflectance simulation), and a layer of $FePS_3$ flake with variable thickness followed by an ultimate layer of air with infinite thickness (see Figure S3 for clarification).

In the simulations, an approximately constant refractive index value of $n$ = 2.45 +i0.15 has been used for $FePS_3$[7], whereas the refractive indices of $SiO_2$ and Si were taken from the database provided in[8]. The approximate value for $FePS_3$ refractive index has been obtained from our previous work[7], where we find this approximation to be enough to be

used in optical contrast simulations to determine the thickness of $FePS_3$ flakes on $SiO_2$ within an estimated error of ±10 nm. The respective $FePS_3$ thicknesses, $t$, according to Figure S3 are: 15, 30, 60, 70, 95 and 105 nm, respectively.

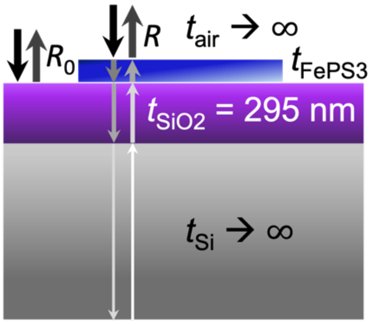


**Figure S3.** Schematic illustration of the multilayered system used in the differential reflectance simulations.

## 3. Quantitative analysis of the fitting parameters obtained from Figure 1 of main manuscript

**Table S1.** Fitting parameters obtained from photoluminescence spectra shown in Figure 1.

| **Sample** | **Peak $X^-$ area** | **Peak $X^0$ area** | **Peak *B* area** | **Area ($X^-$)/ Area ($X^0$)** | **Area ($X^-$)/ Area (*B*)** |
|---|---|---|---|---|---|
| Control | 93.74 | 31.80 | 14.66 | 2.95 | 0.46 |
| Het. | 17.81 | 121.69 | 4.70 | 0.15 | 0.04 |

The photoluminescence spectrum has been fitted to three Lorentzian peaks, corresponding with the negatively charged trion associated to exciton *A* ($X^-$), the neutral exciton associated to exciton *A* ($X^0$) and the exciton *B* (*B*) as follows:

$$f(x) = \frac{a_1}{(x-b_1)^2 + \left({}^{c_1}/_2\right)^2} + \frac{a_2}{(x-b_2)^2 + \left({}^{c_1}/_2\right)^2} + \frac{a_3}{(x-b_3)^2 + \left({}^{c_1}/_2\right)^2} \quad (S1)$$

where the peak area is given by $A = 2\pi\frac{a_i}{c_i}$, $b_i$ denotes the position of the peak and $c_i$ is the full width at half maximum (FWHM) for $i = 1, 2, 3$.

## 4. Estimation of the electron density in one-layer $MoS_2$

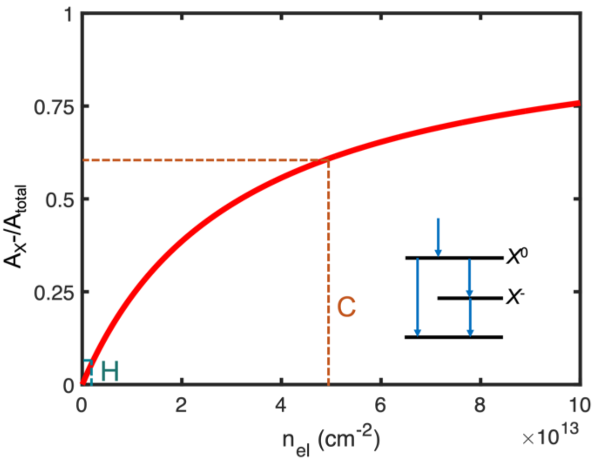


**Figure S4.** Electron density calculated from the mass action law under the assumption of a three-level energy system (sketched in the inset). The orange dashed line indicates the electron density of the control sample (C) whereas the green dashed line corresponds to the electron density of the heterostructure (H).

The mass action law associated with trions is used to evaluate the electron density in 1L $MoS_2$ as part of the van der Waals heterostructure or as a control sample (equation 1 in the main article). Here, the effective mass values are $m_{X^-} = 1.15m_0$, $m_{X^0} = 0.8m_0$ and $m_e = 0.35m_0$, where $m_0$ is the mass of a free electron. The temperature is $T = 300$ K and the trion binding energy is $E_b \sim 30$ meV.

Considering a three-level energy system (inset of Figure S4), the PL intensity of the trion and exciton can be written as[9]

$$A_{X^-} \approx A\gamma_{tr}N_{X^-}, \quad \text{(S2)}$$

$$A_{X^0} \approx A\gamma_{exc}N_{X^0}, \quad \text{(S3)}$$

where $\gamma_{tr}$ and $\gamma_{exc}$ are the radiative decay rates of trions and excitons, respectively.

Substituting expressions S2 and S3, the trion spectral weight is then related to the population of trions and excitons as

$$\frac{A_{X^-}}{A_{total}} = \frac{\frac{\gamma_{tr}}{\gamma_{exc}}\frac{N_{X^-}}{N_{X^0}}}{1+\frac{\gamma_{tr}}{\gamma_{exc}}\frac{N_{X^-}}{N_{X^0}}}, \quad \text{(S4)}$$

where $\gamma_{tr}/\gamma_{exc}$ is equal to $\sim 0.15$[9]. From the mass action model (equation 1 in the main article), the population of trions and excitons is directly related to the electron density as

$$\frac{N_{X^-}}{N_{X^0}} = \frac{n_{el}}{\left(\frac{4m_{X^0}m_e}{\pi\hbar^2 m_{X^-}}\right)k_B T\exp\left(-\frac{E_b}{k_B T}\right)} \approx 2.096\times 10^{-13}\, n_{el}\,(\text{cm}^2). \quad \text{(S5)}$$

Thus, the trion spectral weight varies with the electron density as (plotted in Figure S4)

$$\frac{A_{X^-}}{A_{\text{total}}} \approx \frac{3.14 \times 10^{-14}\, n_{\text{el}}}{1 + 3.14 \times 10^{-14}\, n_{\text{el}}}. \qquad \text{(S6)}$$

## 5. Analysis of the photoluminescence of 1L $MoS_2/FePS_3$ heterostructures with different $FePS_3$ thickness

The photoluminescence spectra at room temperature of all the samples have been fitted to three Lorentzian peaks, corresponding with the negatively charged trion associated to exciton $A$ ($X^-$), the neutral exciton associated to exciton $A$ ($X^0$) and the exciton $B$ ($B$) as follows:

$$f(x) = \frac{a_1}{(x-b_1)^2 + \left({}^{c_1}/_{2}\right)^2} + \frac{a_2}{(x-b_2)^2 + \left({}^{c_1}/_{2}\right)^2} + \frac{a_3}{(x-b_3)^2 + \left({}^{c_1}/_{2}\right)^2} \quad \text{(S7)}$$

where the peak area is given by $A = 2\pi \frac{a_i}{c_i}$, $b_i$ denotes the position of the peak and $c_i$ is the full width at half maximum (FWHM) for $i = 1, 2, 3$.

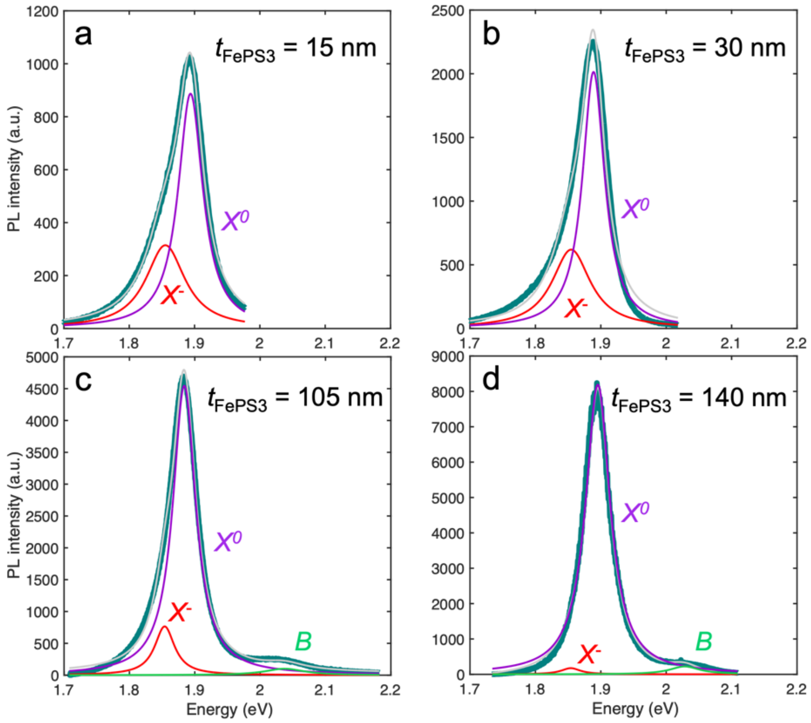


**Figure S5. (a) – (d)** Photoluminescence spectra of several 1L $MoS_2/FePS_3$ heterostructures with different thicknesses of $FePS_3$ ($t_{FePS3}$), where the spectral weight between trion ($X^-$) and neutral exciton ($X^0$) reveals the amount of charge transferred at the heterointerface.

♦ Heterostructures:

**Tables S2.** Relation between the thickness of underneath $FePS_3$ flakes and the corresponding ratio for the trion/exciton peak areas and its estimated charge transfer. The control samples used as reference for each estimate are found in the second table below.

| **$FePS_3$ thickness (nm)** | $A_{X^-}/A_{X^0}$ | **Charge transfer (%)** |
|---|---|---|
| 15 | 0.5622 | 81(1) |
| 30 | 0.5322 | 82(1) |
| 70 | 0.4408 | 86(1) |
| 95 | 0.3125 | 93(2) |
| 105 | 0.1464 | 95(1) |
| 140 | 0.0169 | 99(3) |

♦ Controls:

| **Sample ($MoS_2/SiO_2$)** | $A_{X^-}/A_{X^0}$ |
|---|---|
| Control (1) | 2.9489 |
| Control (2) | 4.5580 |
| Control (3) | 2.3306 |

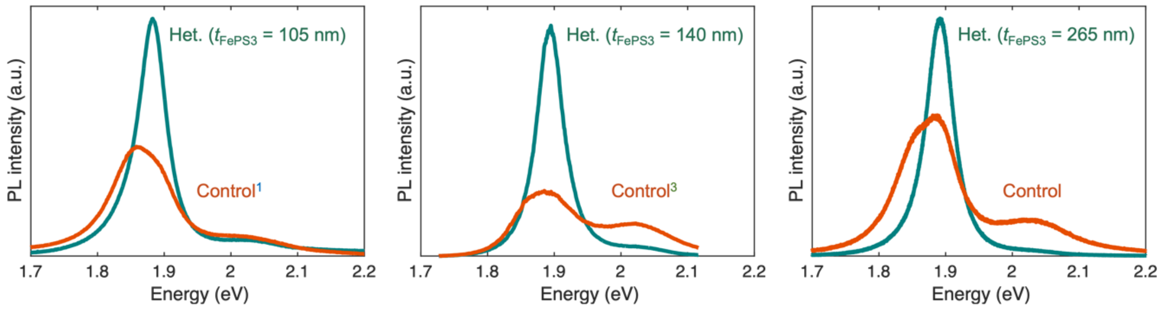


**Figure S6.** Photoluminescence enhancement in single-layer $MoS_2/FePS_3$ heterostructures with thicknesses of $FePS_3$ larger than 100 nm.

## 6. Valence band UPS spectrum of bulk $FePS_3$

From a linear fit to the data in the valence band region of the UPS spectrum of bulk $FePS_3$, we obtain an energy cut-off of ~0.56 eV. Considering an error in the determination of the UPS slope of ±0.2 eV, and taking into account the activation energy obtained in section S8, the difference between both energies falls within the permitted uncertainty range.

Considering minor calculation error in the low-temperature photocurrent measurements, we have placed the valence band maximum ~0.4 eV below the Fermi level of $FePS_3$ (Figure 2b of main article).

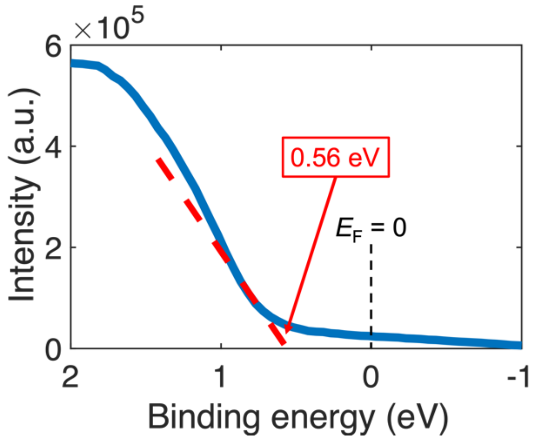


**Figure S7.** Valence band cut-off of UPS spectrum of bulk $FePS_3$.

## 7. Kelvin probe force microscopy

KPFM measurements were performed using a TiN-coated conductive tip (FMG01 from NT-NDT). The tip work function was calibrated against a freshly exfoliated HOPG graphite crystal and calculated from the measured contact potential difference ($\Delta V_{cpd} \sim$ 0.3eV) and the known work function of HOPG (4.6 eV) as $\phi_{tip} = \phi_{HOPG} + \Delta V_{cp} \sim 4.9$ eV. Thus, the work function of a freshly exfoliated crystal of $FePS_3$ was obtained as $\phi_{FePS3} = \phi_{tip} - \Delta V_{cp} \sim 5.1$ eV.

## 8. Thermal activation energy of a multi-layer $FePS_3$ flake

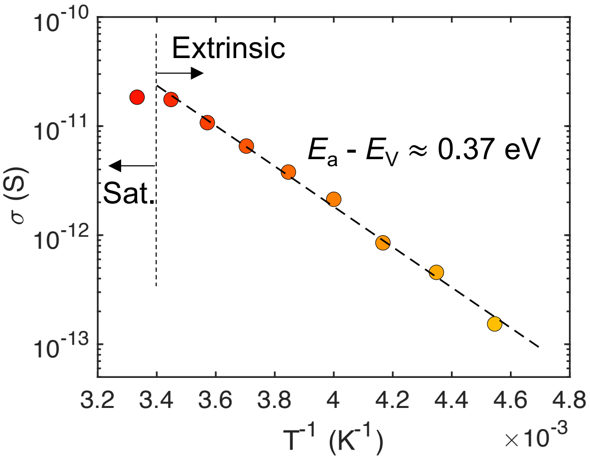


**Figure S8.** Arrhenius plot of conductance as a function of the inverse temperature of a several-layer $FePS_3$ flake.

The activation energy of a multi-layer $FePS_3$ flake, in contact with prepatterned Ti/Au electrodes, has been obtained through temperature-dependent transport measurements. Conductivity in *p*-type semiconductors, as for the case of $FePS_3$, is mediated by holes that are available in the valence band due the promotion of electrons to higher acceptor energy levels. These acceptor levels are generated by the 3d levels of $Fe^{+2}$ ions that are partially filled[10]. Here, the extrinsic behavior[11] of $FePS_3$ has been studied as a function of the applied source-drain voltage for a temperature range between 220 to 285 K. In this range, the conductivity is a thermally activated process of Arrhenius-type[12] where thermal energy favors the formation of electron-hole pairs, promoting conductivity through holes in the material. We found that below 220 K the current is so small that this one is masked by the noise of the measurement itself. Above 285 K, there is a saturation range where all the valence band electrons have been promoted to the acceptor energy levels and the conductivity exhibit a different behavior as pointed out by Kuzminskii *et al*[13]. The Arrhenius-type equation for conductivity as a function of temperature is given by

$$\sigma \propto e^{-\frac{(E_a - E_V)}{K_B T}} \quad (S8)$$

where $\sigma$ is the conductivity, $E_a$ is an activation energy, $E_V$ is the valence band edge energy, $K_B$ is the Boltzmann's constant and $T$ is the temperature. A linear fit to the data falling within the extrinsic thermal range (from 220 K to 285 K) reveals an energy difference of $(E_a - E_V) \sim 0.37$ eV $\pm$ 0.02 eV.

## 9. Theoretical analysis of $MoS_2/FePS_3$

**Electronic structure of $MoS_2/FePS_3$:** The electronic structure was calculated considering 1L $MoS_2$ and 1L of $FePS_3$, introducing the zigzag antiferromagnetic configuration characteristic of $FePS_3$ at 0 K. The results show the valence and conduction bands are very close to the electronic structure of both materials separately, pointing out the lack of hybridization between both systems. The electronic gap corresponds to the 1.23 eV bandgap of $FePS_3$. $MoS_2$ and $FePS_3$ were simulated separately to calculate the work function and the electronic energies were scaled respect to the vacuum. To properly simulate the bulk $FePS_3$ we prepared slabs of different size to converge the work function. We found that a slab of 4 layers isolated by vacuum provides essentially the same results that considering higher number of layers. Both the work functions and the energy levels are represented in Figure S10.

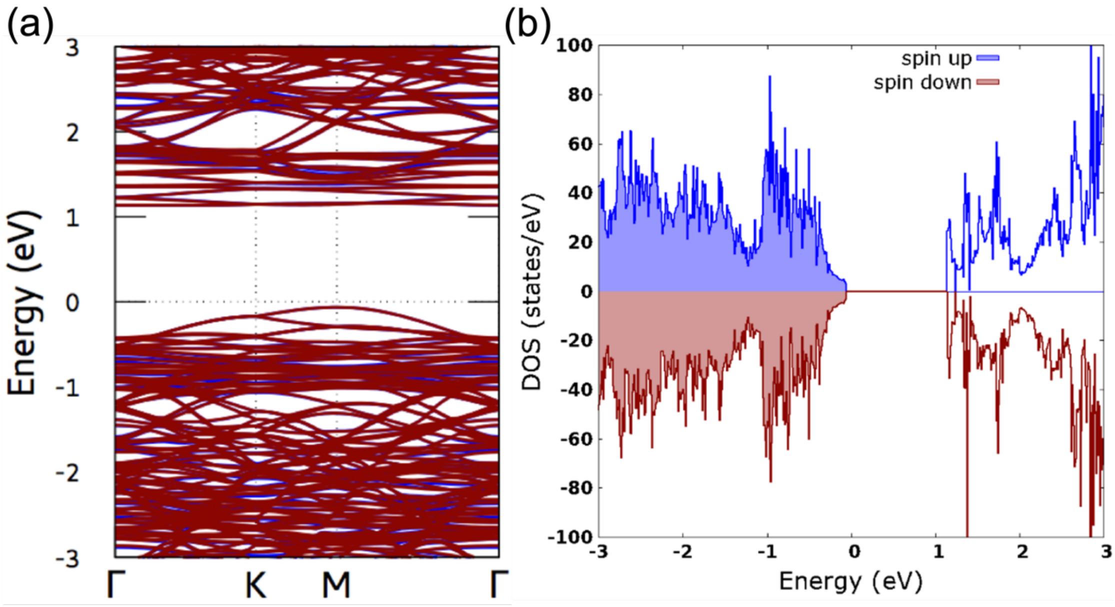


**Figure S9.** DFT simulation of 1L MoS2/ML $FePS_3$ **(a)** electronic band structure **(b)** density of states.

**Band alignment:**

The work function was first determined for $MoS_2$ and $FePS_3$ monolayers and bulk $FePS_3$, which was simulated with slabs formed by 4 and 6 layers, being already converged in the 4-layers slab calculation. Our DFT results yield work functions of 5.22 eV and 5.11 eV for $FePS_3$ and $MoS_2$, respectively, with the Fermi energy of $MoS_2$ lying slightly above the Fermi energy of $FePS_3$ (Figure S10). We also determine the work function of single-layer $FePS_3$ ( $\phi$ = 5.25 eV), which indicates that the same type I of band alignment would be preserved at the 2D limit (Figure S11).

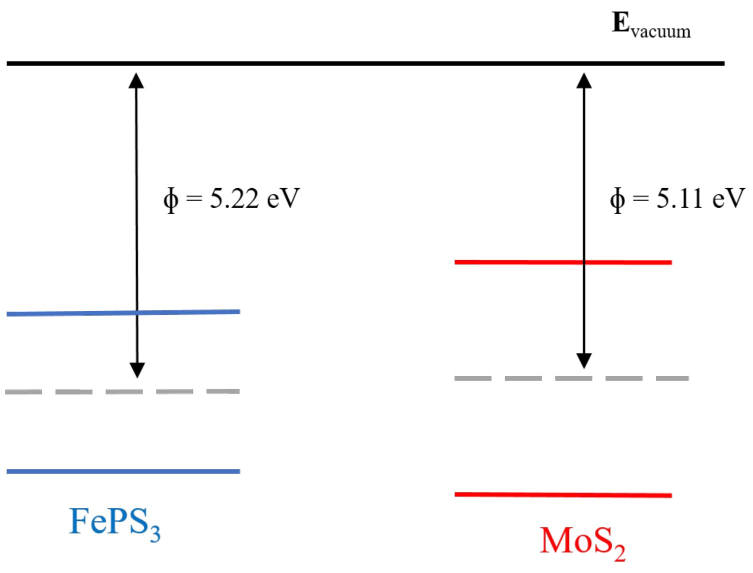


**Figure S10.** Band alignment diagram of $FePS_3$ (4 layers) and 1L $MoS_2$.

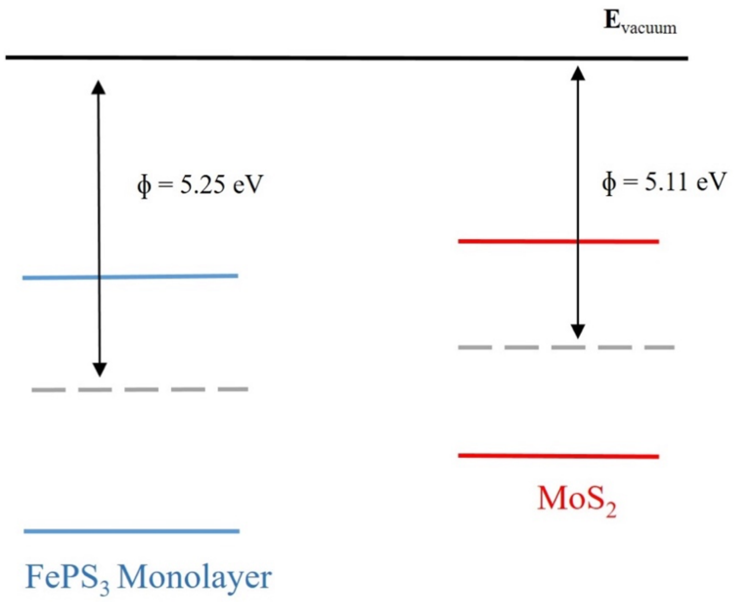


**Figure S11.** Band alignment diagram of 1L $FePS_3$ and 1L $MoS_2$.

**Bader analysis:** As presented in the main text, charge transfer calculations indicate there is no presence of charge density moving along the interface of a natural, non-doped heterostack. A Bader analysis was performed to corroborate our findings. This analysis confirms an important redistribution along both systems, being the S atoms the acceptors of charge. However, as indicated in this table, the net charge moving outside the layers is almost zero, probing the inexistent charge transfer between $MoS_2$ and $FePS_3$ in absence of doping.

**Table S3.** Bader analysis of $MoS_2$-$FePS_3$ heterostructure. Atomic charge variations are expressed respect to the charge included in the pseudopotentials. Positive sign corresponds to a depletion of electrons.

| | **Fe** | **P** | **S** | **Mo** | **S** |
|---|---|---|---|---|---|
| $e^-$ transfer per atom | 1.095 | 1.052 | -0.714 | 1.116 | -0.560 |
| $e^-$ transfer in the material (per cell) | 0.044 | | | -0.048 | |

## 10. Theoretical analysis of S vacancy in $MoS_2$

To give an explanation to the unusual charge transfer observed in the experiment, the existence of sulfur vacancies was proposed. A 4x4 supercell was constructed to ensure the absence of interaction between different defects originated in the periodical boundary conditions generated in the DFT simulation. In this conditions, one atom of sulfur was removed to simulate the vacancy. The electronic structure of this system is presented in Figure S12.

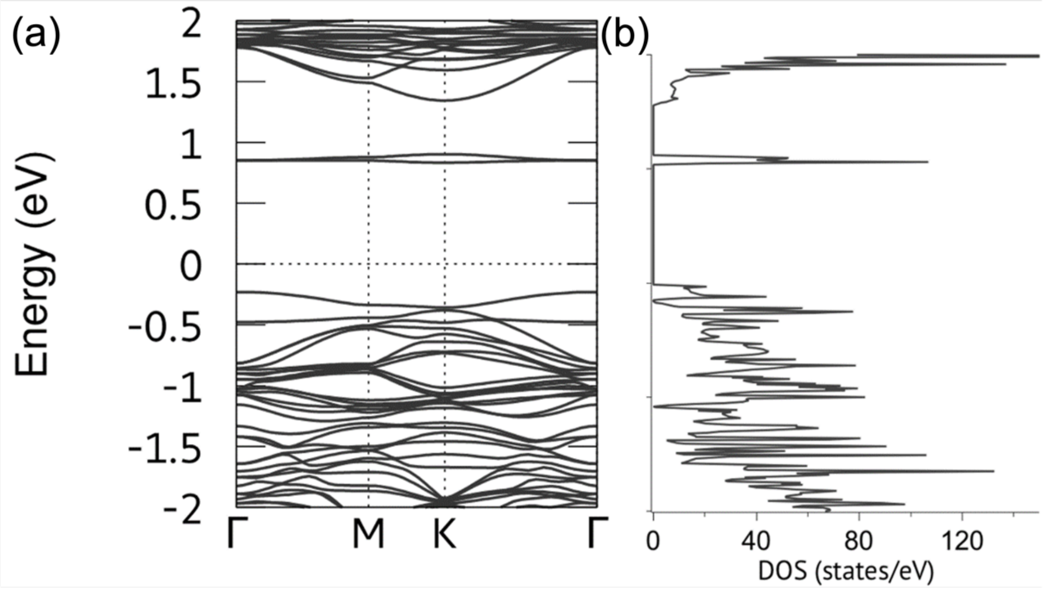


**Figure S12.** Electronic structure of vacant $MoS_2$. (a) electronic band structure (b) density of states. A localized state corresponding to the sulfur vacancy is present in the electronic structure of $MoS_2$.

**Band alignment:** To analyze the effect of this vacancy in the band alignment we calculated the work function and scaled the energy levels to the vacuum. The Fermi level lies on the energy level of the defect (Figure S13).

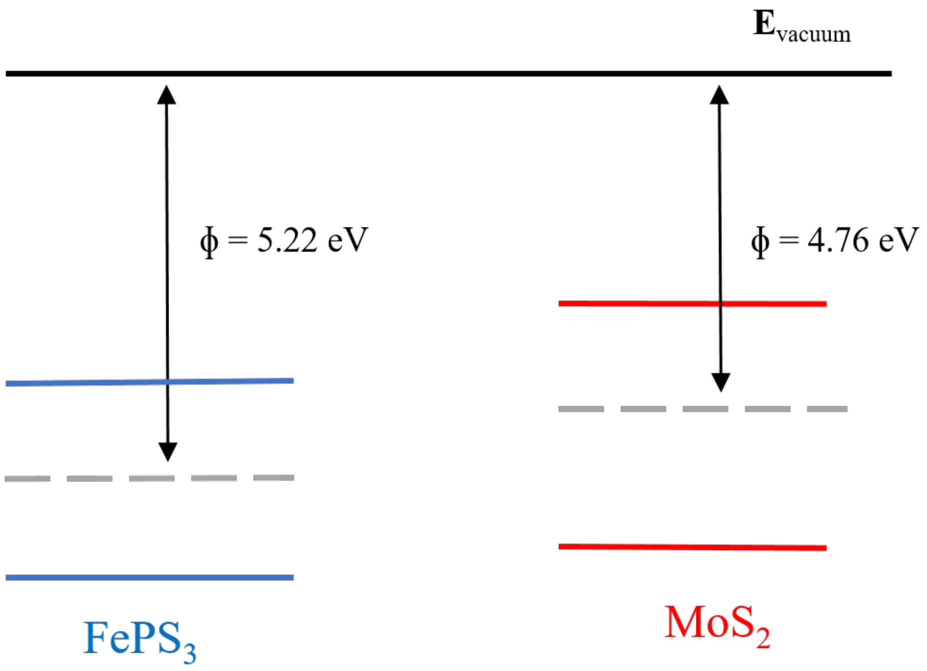


**Figure S13.** Band alignment diagram of ML $FePS_3$ (4 layers) and 1L $MoS_2$ (4x4 supercell in presence of 1 sulfur atom vacancy). The energy levels of $MoS_2$ increase they energy providing a work function of 4.76 eV, very close to the experimental findings.

### 11. Photoluminescence in heterostructures prepared in air

To discern the effects of sample preparation under a controlled atmosphere and in air conditions, new heterostructures were fabricated in air. Figure S14 shows the photoluminescence spectra of control and heterostructure samples prepared in air and fitted to three Lorentzian peaks (negatively charged exciton $X^-$, neutral exciton $X^0$ and exciton $B$).

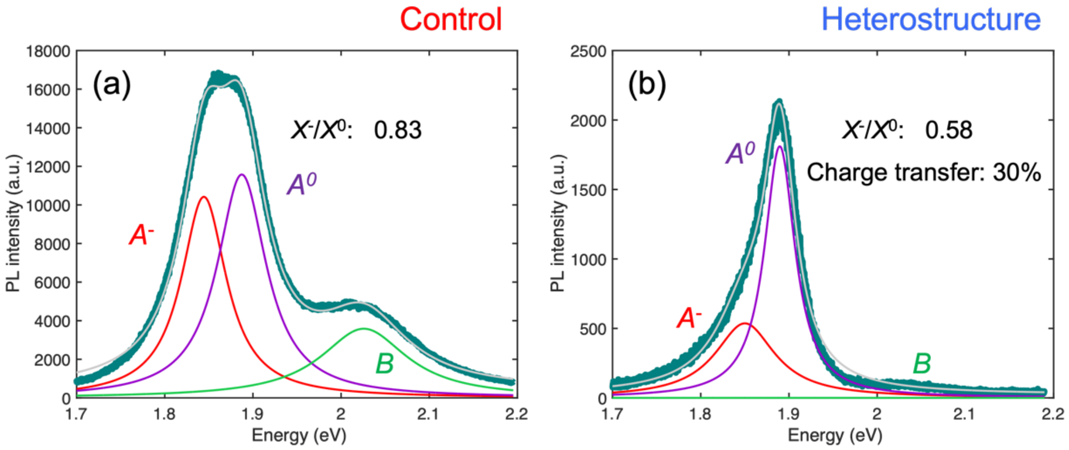


**Figure S14**. **(a)** Photoluminescence spectrum of a control sample prepared in air. **(b)** Photoluminescence spectrum of a heterostructure prepared in air with a $FePS_3$ flake of 40 to 50 nm thickness.

The first observation is the highly quenched photoluminescence of the heterostructure compared to the control sample, in contrast with the heterostructures prepared under controlled atmosphere. The second observation is a decreased charge transfer for heterostructures prepared in air than under controlled conditions. For instance, a charge transfer of 30% is achieved in the heterostructure of Figure S14, containing a $FePS_3$ flake with thickness between 40-50 nm. Recalling the charge transfer vs. $FePS_3$ thickness guideline of Figure 2d, a charge transfer of about 82% should be achieved for the same heterostructure fabricated under controlled atmosphere. From these observations, we conclude that charge transfer in these heterostructures plays a major role in the photoluminescence emission, discarding or leaving in second place possible energy transfer mechanisms.

## 12. Exciton lifetime characterization

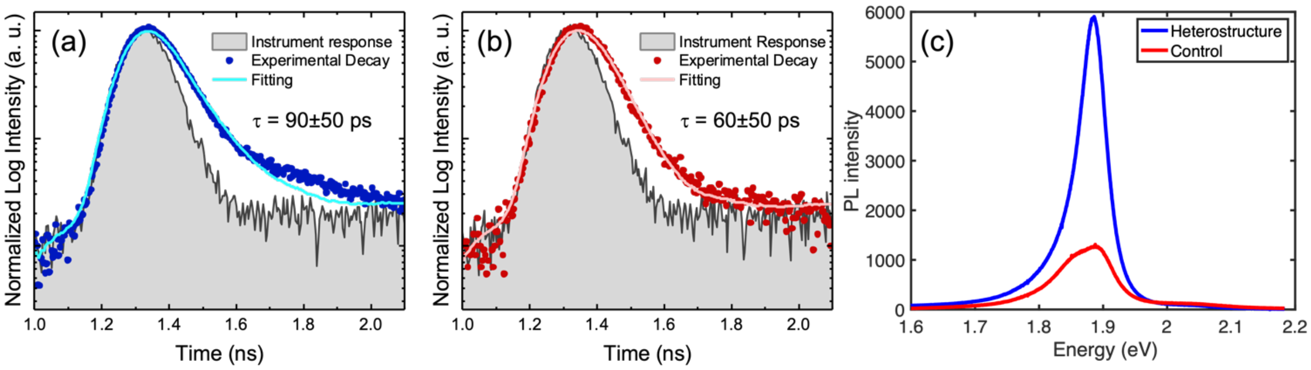


**Figure S15. (a) – (b)** Experimentally time-resolved PL of 1L $MoS_2/FePS_3$ heterostructure and $MoS_2$ on $SiO_2$, respectively, at room temperature. A single exponential decay fit to the data results in a radiative lifetime of 90 ps and 60 ps, respectively. **(c)** PL of both samples at room temperature.

**Time-resolved photoluminescence.** TRPL measurements were carried out using a home-made optical setup operating at room temperature. As an excitation source, we used a 1034-nm Flint FL1 laser with 80 MHz repetition rate and a pulse duration of <120 fs connected to a free-standing harmonic generator, which converts the initial beam to 517 nm wavelength. The laser power is filtered by using a variable metallic neutral density filter, and then is reflected on a dichroic mirror that directs light to a high numerical aperture lens. In this manner, the final laser power reaching the sample is adjusted to a variable power below 100 μW to rule out any possibility of laser-induced damage. The detection of the PL signal was performed by means of a silicon CCD attached to a double-exit spectrometer. A photomultiplier detector is connected at the second exit of the spectrometer and monitored by means of a Time Correlated Single Photon Counting electronics.

## 13. Four Lorentzian peak fitting of photoluminescence spectrum at low temperature

The photoluminescence spectra as a function of temperature have been fitted to four Lorentzian peaks, accounting for the bands *D* (associated to defects), negative trion ($X^-$), neutral exciton *A* ($X_0$) and exciton *B* (*B*) following the expression below:

$$f(x) = \frac{a_1}{(x-b_1)^2 + \left({}^{c_1}/_2\right)^2} + \frac{a_2}{(x-b_2)^2 + \left({}^{c_1}/_2\right)^2} + \frac{a_3}{(x-b_3)^2 + \left({}^{c_1}/_2\right)^2} + \frac{a_4}{(x-b_4)^2 + \left({}^{c_4}/_2\right)^2} \qquad (S9)$$

where the peak area is given by $A = 2\pi \frac{a_i}{c_i}$, $b_i$ denotes the position of the peak and $c_i$ is the full width at half maximum (FWHM) for $i = 1, 2, 3$.

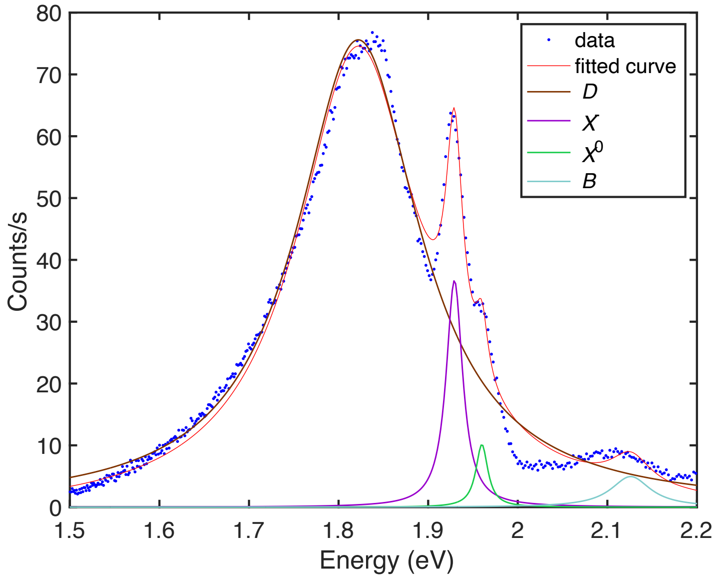


**Figure S16.** Photoluminescence spectrum of a 1L $MoS_2/FePS_3$ heterostructure at 10 K, fitted to four Lorentzian peaks.

Although we do perform the fitting including band *B*, in the variable temperature measurements, we unfortunately had to ignore the quantitative analysis of this peak because in this experimental setup the pump laser tails at this position, and thus the fittings of the *B* band are disturbed by this signal.

## 14. Semiconductor bandgap model

To quantify the blue shifting of the PL emission in the heterostructure and control samples when decreasing temperature, a standard semiconducting bandgap model has been used[14]:

$$E_{\mathrm{g}}(T) = E_{\mathrm{g}}(0) - S\hbar\omega\left[\coth\left(\frac{\hbar\omega}{2k_BT}\right) - 1\right], \qquad (S10)$$

where $E_{\mathrm{g}}(0)$ is the bandgap at 0 K, $S$ is a parameter related to the electron-phonon coupling strength and $\hbar\omega$ is the average phonon energy involving the electron-phonon interaction. This model perfectly fits the temperature dependence of the three peaks labeled as $D$, $X^-$ and $X^0$ in Figure 3c (heterostructure) and 3f (control sample).

**Table S4.** Summary of the parameters used to fit the PL spectra of Figure 4a-b into the model described in Equation 4.

| | Peak | $E_{\mathrm{g}}$ (0) (eV) | $S$ (a.u.) | $\hbar\omega$ (meV) |
|---|---|---|---|---|
| Heterostructure | $D$ | 1.821 | 7.488 | 10.27 |
| | $X^-$ | 1.929 | 1.817 | 17.26 |
| | $X^0$ | 1.959 | 2.491 | 31.74 |
| Control | $D$ | 1.831 | 5.019 | 13.24 |
| | $X^-$ | 1.924 | 1.782 | 23.18 |
| | $X^0$ | 1.960 | 2.093 | 29.23 |

## 15. Activation energies of sulfur vacancies in heterostructure and control samples

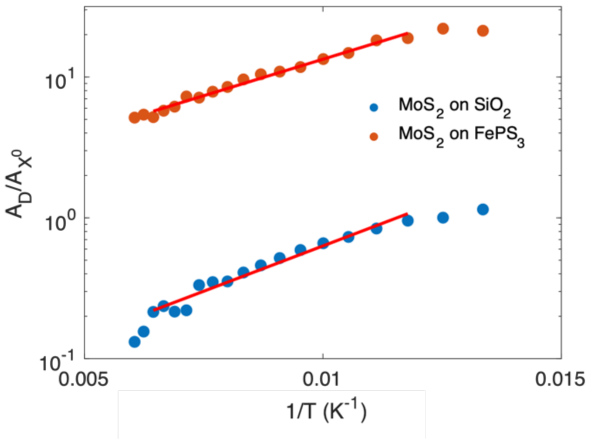


**Figure S17.** Temperature dependence of the defect peaks relative spectral weight for $MoS_2$ on $SiO_2$ and on top of $FePS_3$. The red continuous lines represent the fit of the data to an Arrhenius model. From its slope, activation energies of 11 meV and 9 meV are obtained for $MoS_2$ on $SiO_2$ and on $FePS_3$, respectively.

## References


1. Li, H., *et al*. From bulk to monolayer $MoS_2$: evolution of Raman scattering. *Advanced Functional Materials* **2012**, *22*, 1385.
2. Scagliotti, M., *et al*. Raman scattering in antiferromagnetic $FePS_3$ and $FePSe_3$ crystals. *Physical Review B* **1987**, *35*, 7097.
3. Wang, X., *et al*. Raman spectroscopy of atomically thin two-dimensional magnetic iron phosphorus trisulfide ($FePS_3$) crystals. *2D Materials* **2016**, *3*, 031009.
4. Du, K. Z., *et al*. Weak van der Waals stacking, wide-range band gap, and Raman study on ultrathin layers of metal phosphorus trichalcogenides. *ACS Nano* **2016**, *10*, 1738.
5. Lee, J. U., *et al*. Ising-type magnetic ordering in atomically thin $FePS_3$. *Nano Letters* **2016**, *16*, 7433.
6. Byrnes, S. J. Multilayer optical calculations. *ArXiv preprint* **2016**, 1603.02720.
7. Ramos, M., *et al*. Ultra-broad spectral photo-response in $FePS_3$ air-stable devices. *npj 2D Materials and Applications* **2021**, *5*, 1.
8. Palik E. *Handbook of Optical Constants of Solids* **1985**, 1, 759.
9. Mouri, S., *et al*. Tunable photoluminescence of monolayer $MoS_2$ via chemical doping. *Nano Letters* **2013**, *13*, 5944.
10. Grasso, V., *et al*. Conduction processes in the layered semiconductor compound $FePS_3$. *Physical Review B* **1990**, 42, 1690.
11. Foot, P. J. S., *et al*. Optical and electronic properties of the layered semiconductors $NiPS_3$ and $FePS_3$. *Materials Research Bulletin* **1980**, 15, 189.
12. Haines, C. R. S., *et al*. Pressure-induced electronic and structural phase evolution in the van der Waals compound $FePS_3$. *Physical Review Letters* **2018**, 121, 266801.
13. Kuzminskii, Y. V., *et al*. Iron and nickel phosphorus trisulfides as electroactive materials for primary lithium batteries. *Journal of Power Sources* **1995**, 55, 133.
14. O'donnell, K. P.; Chen, X. Temperature dependence of semiconductor band gaps. *Applied Physics Letters* **1991**, *58* (25), 2924-2926.
